# The *Xenopus laevis* Atg4B Protease: Insights into Substrate Recognition and Application for Tag Removal from Proteins Expressed in Pro- and Eukaryotic Hosts

**DOI:** 10.1371/journal.pone.0125099

**Published:** 2015-04-29

**Authors:** Steffen Frey, Dirk Görlich

**Affiliations:** Abteilung Zelluläre Logistik, Max-Planck-Institut für Biophysikalische Chemie, Göttingen, Germany; Russian Academy of Sciences, Institute for Biological Instrumentation, RUSSIAN FEDERATION

## Abstract

During autophagy, members of the ubiquitin-like Atg8 protein family get conjugated to phosphatidylethanolamine and act as protein-recruiting scaffolds on the autophagosomal membrane. The Atg4 protease produces mature Atg8 from C-terminally extended precursors and deconjugates lipid-bound Atg8. We now found that *Xenopus laevis* Atg4B (xAtg4B) is ideally suited for proteolytic removal of N-terminal tags from recombinant proteins. To implement this strategy, an Atg8 cleavage module is inserted in between tag and target protein. An optimized xAtg4B protease fragment includes the so far uncharacterized C-terminus, which crucially contributes to recognition of the *Xenopus* Atg8 homologs xLC3B and xGATE16. xAtg4B-mediated tag cleavage is very robust in solution or on-column, efficient at 4°C and orthogonal to TEV protease and the recently introduced proteases bdSENP1, bdNEDP1 and xUsp2. Importantly, xLC3B fusions are stable in wheat germ extract or when expressed in *Saccharomyces cerevisiae*, but cleavable by xAtg4B during or following purification. We also found that fusions to the bdNEDP1 substrate bdNEDD8 are stable in *S*. *cerevisiae*. In combination, or findings now provide a system, where proteins and complexes fused to xLC3B or bdNEDD8 can be expressed in a eukaryotic host and purified by successive affinity capture and proteolytic release steps.

## Introduction

Macroautophagic self-degradation (hereafter autophagy) is a common response of eukaryotic cells to stress stimuli like starvation or pathogen infection [1,2]. Generally, bulk cytoplasm is non-selectively enclosed in autophagosomes, which are double membrane vesicles that fuse with lysosomes or the vacuole for degradation or recycling of the engulfed components. However, also specific targets can be degraded via receptors and adaptor proteins [3–5]. During autophagosome formation, small ubiquitin-like proteins (UBLs) of the Atg8 family are covalently attached via their C-terminal Gly residue to phosphatidylethanolamine (PE) lipids on the autophagosomal membrane [6]. Although it is clear that Atg8 lipidation and tethering to the autophagosomal membrane is essential for autophagosome biogenesis, the precise mechanism of Atg8 function so far remains elusive [2,7,8].

Unlike *S*. *cerevisiae*, which only has one Atg8 paralog, mammals encode two subfamilies of Atg8-like proteins (GABARAP/GATE16 and LC3). These subfamilies often comprise several members and act in distinct steps of autophagosome formation [9–14]. All Atg8 family members are structurally similar. Their structured core domain consists of an ubiquitin-like β-grasp fold preceded by two additional N-terminal α-helices [15–19]. This domain represents a versatile protein interaction surface, which is essential for recruitment of the autophagy machinery to the autophagosomal membrane [2]. The characteristic and flexible C-terminus of all Atg8 family members ends with Phe-Gly (FG) or Tyr-Gly (YG). It is generated by Atg4 proteases that cleave C-terminally extended precursors [20–25]. This group of highly specific proteases is also responsible for deconjugating Atg8 proteins from PE [23], a process that is required at a late stage of autophagosome formation [12,26].

As for Atg8, several paralogous Atg4-like proteases exist in higher eukaryotes, which might have different specificities for individual Atg8 paralogs [27,28]. Amongst the four human Atg4 paralogs (Atg4A-D [20,22,24,25]), Atg4B is the most versatile and active protease on recombinant fusion proteins. It can process the human Atg8 paralogs LC3B, GATE16, GABARAP and Atg8L with similar efficiencies [27]. The other three Atg4 enzymes are catalytically much less active.

Atg8-like proteins represent only one class of UBLs. This group of small protein modifiers also includes the founding member ubiquitin, SUMO and NEDD8, which all act as regulators of various intracellular processes (reviewed in references [29,30]). In contrast to Atg8-like proteins, however, other UBLs generally possess a C-terminal Gly-Gly (GG) motif and are conjugated to proteins via isopeptide bonds formed between their C-terminal carboxyl and group primary amine groups on the surface of target proteins. Importantly, all mentioned UBLs are initially processed and often deconjugated by dedicated proteases [29]. In most cases, these proteases are highly specific and efficient, and are therefore ideally suited for biochemical applications.

The yeast SUMO specific protease Ulp1p (scUlp1; [31]), for example, is used for the *in vitro* tag-removal from recombinant proteins [32]. Recently, we characterized additional UBL-specific proteases and found that the *Brachypodium distachyon* (bd) SUMO- and NEDD8-specific proteases bdSENP1 and bdNEDP1 remove tags even more robustly than scUlp1 and up to 1000 times more efficiently than TEV protease. Both enzymes can thus be used for the highly efficient purification of recombinant proteins by on-column or post-column cleavage [33,34]. Importantly, bdSENP1 and bdNEDP1 display mutually exclusive (i.e. orthogonal) substrate specificity, which can be exploited for purification of protein complexes with a defined subunit stoichiometry by cycles of tag-mediated affinity capture and subsequent proteolytic release [34].

While this method is straightforward when purifying proteins or protein complexes expressed in prokaryotic hosts, UBL fusions are typically cleaved in eukaryotic hosts by endogenous UBL-processing enzymes. Recently, the SUMO variant SUMOstar has been introduced, which allows purification of recombinant fusion proteins also from eukaryotic hosts [35,36]. Further UBL-like protease substrates, which are stable in eukaryotic hosts but readily cleavable by dedicated proteases, might become valuable tools for the purification of protein complexes expressed in eukaryotes. In other applications, such substrate/protease pairs may be used for specific *in-vivo* manipulations of recombinant fusion proteins: If UBL fusions can be stably expressed in a given host cell, cleavage can be induced upon co-expression of the respective protease. Such systems can, e.g., be applied to control the stability or localization of a protein of interest [37–39].

Recently we also described the application of the *S*. *cerevisiae* (sc) Atg4 protease for tag removal [33]. scAtg4 is highly active *in vitro* and displays mutually exclusive cleavage specificity to SUMO, NEDD8 and ubiquitin-processing enzymes. Yet, neither this protease nor scAtg8 fusion proteins perform well in terms of solubility and/or expression level.

We now identified a seemingly optimal alternative, namely the Atg4B protease from *Xenopus laevis* (xAtg4B), along with its substrates xLC3B and xGATE16. N-terminal xLC3B- or xGATE16-containing tags allow for high yield and soluble expression of target proteins. Likewise, the production of recombinant xAtg4B protease is straightforward. We found compelling evidence that the so far uncharacterized C-terminal extension of xAtg4B contributes to the recognition of substrates containing xLC3B or xGATE16. The optimal enzyme fragment xAtg4B^14-384^ is extraordinary robust: It cleaves its substrates also at low temperatures, in the presence of high salt or in problematic sequence contexts. At 0°C, xAtg4B has a turnover rate similar to bdNEDP1 and is thus ≈30 to 50 times more active than TEV protease [33]. Additionally, it has orthogonal specificity to the recently introduced bdSENP1 and bdNEDP1 proteases. Importantly, we also show that xLC3B fusions are stable in *S*. *cerevisiae* and in wheat germ extract. Likewise, we found bdNEDD8 fusions to be resistant towards endogenous *S*. *cerevisiae* proteases. Thus, the bdNEDD8 and xLC3B cleavage modules allow purifying recombinant target proteins by the affinity capture and proteolytic release strategy also from selected eukaryotic hosts.

## Methods

### Protein sequence alignments

Sequence alignments were performed using the ClustalW algorithm implemented in MegAlign version 11.2.1. (DNAStar, Inc.).

### Protein expression and purification

*Substrate proteins and proteases* were over-expressed in *E*. *coli* from appropriate expression vectors (S1 Table) and purified as described before [33].

### Cleavage assays

*Cleavage assays in solution and on column* were performed as described before [33,34]. If not stated otherwise, all assays were performed in LS-S buffer (250 mM NaCl, 40 mM Tris/HCl pH 7.5, 2 mM MgCl_2_, 250 mM sucrose, 2 mM DTT). On-column cleavage assays were done on silica- or Sepharose-based Ni^2+^ chelate resins with high porosity.

### Estimation of apparent cleavage rates

For a more quantitative comparison of cleavage conditions and protease variants, we wanted to compare cleavage rates. The cleavage reactions should not follow a simple Michaelis-Menten kinetics. Instead, they are complicated by the fact that one of the end products, namely the cut off xLC3B or xGATE16 module, binds strongly to the protease and thus exhibits product inhibition. Given, however, that this product and the substrate have a very large and essentially identical interaction interface with the protease, it is reasonable to assume that the two bind with approximately the same affinities. As detailed below, such assumption simplifies the time course of the cleavage reaction such that it can be approximated by a single exponential and that a single time point becomes sufficient to derive a cleavage rate.

For a simple cleavage reaction [*S*]→[*P*]+[*P*'] with only product [*P*] competing with the substrate [*S*] for the enzyme [*E*], we can extend the Michaelis-Menten equation [40] as

$$v=-\frac{d[S]}{dt}=\frac{k_{cat}\cdot [E_{0}]\cdot [S]}{K_{M}(1+\frac{\left[P\right]}{K_{P}}+\frac{\left[S\right]}{K_{M}})}$$ (eq 1)

where [*E*_*0*_] is the total enzyme concentration, *K*_*M*_ is the Michaelis-Menten constant and *K*_*P*_ is the end product inhibition constant. We assume that substrate and product have similar affinity for the enzyme (i.e. *K*_*M*_ = *K*_*P*_). We can thus simplify eq 1 to

$$v=-\frac{d[S]}{dt}=\frac{k_{cat}\cdot [E_{0}]\cdot [S]}{K_{M}+[P]+[S]}$$ (eq 2)

Because during the cleavage reaction one molecule of product is formed per substrate molecule, we can further substitute [*P*] = [*S*_*0*_]-[*S*], with S_*0*_ being the initial substrate concentration:

$$v=-\frac{d[S]}{dt}=\frac{k_{cat}\cdot [E_{0}]}{K_{M}+[S_{0}]}\cdot [S]$$ (eq 3)

With

$$k_{app}=\frac{k_{cat}\cdot [E_{0}]}{K_{M}+[S_{0}]}$$ (eq 4)

we can derive the following single exponential

$$\frac{\left[S\right]}{\left[S_{0}\right]}=1-e^{-k_{app}\cdot t}$$ (eq 5)

For a comparison of cleavage rates from single time points, we compare the apparent cleavage rates *k*_*app*_ without explicitly determining *K*_*M*_ and *k*_*cat*_.

### Dynamic light scattering (DLS)

Proteases diluted to 10 μM in LS-S buffer were ultracentrifuged (200.000 g, 30 min) and assayed in a closed cuvette using a DynaPro NanoStar DLS instrument (Wyatt Technology). To acquire heat denaturation curves, the temperature was automatically raised by 1°C every 10 min. DLS signals were acquired just before each temperature step.

### *In vitro* binding assays

A Ni^2+^ chelate resin was loaded with 40 μM His_14_-Spacer-xLC3B-GFP or His_14_-Spacer-xGATE16-GFP. An empty resin served as a control. 20 μl aliquots were incubated with 100 μl of an equimolar mixture of full-length protease and a protease fragment (10 μM each) for 1 h at 25°C in LS-S buffer. After washing (3x 30 sec), bound proteins were eluted with SDS sample buffer containing 500 mM imidazole and analyzed by SDS-PAGE and Coomassie staining.

### Example purifications from *E*. *coli*

Relevant fusion proteins were over-expressed from appropriate expression vectors in *E*. *coli* (S1 Table). Cleared lysates in LS buffer (280 mM NaCl, 45 mM Tris/HCl pH 7.5, 4.5 mM MgCl_2_, 10 mM DTT) + 15 mM imidazole were incubated with a Ni^2+^ chelate resin. After washing, the target proteins were eluted with 500 nM xAtg4B^14-384^ in LS buffer at 4°C. After 1 h, proteins remaining on the resin were eluted with 0.5 M imidazole in LS buffer. Relevant fractions were analyzed by SDS-PAGE. Samples taken during elution were additionally quantified by measuring the OD_280_.

### Substrate stability in eukaryotic extracts

1 μM of protease substrates containing MBP as a target protein were incubated with 10 μl of indicated lysates in the presence or absence of a protease mix containing scUlp1, SUMOstar protease [35,36], xAtg4B^14-384^ and trAtg4B (0.1 μM each) for 2 h at 25°C in 12.5 μl total volume. Reaction products were analyzed by Western blot with an antibody recognizing *E*. *coli* MBP (Sigma-Aldrich #M1321; 1:5000 dilution) and a goat anti-mouse secondary antibody coupled to IRDye800CW (LI-COR #926–32210; 1:5000 dilution). Blots were scanned with an Odyssey infrared imaging system (LI-COR).

### Yeast expression

*S*. *cerevisiae* strain SFY122 (S288C, Mat*α*, H2B-CFP::TRP1, *his3Δ200*, *leu2Δ0*, *lys2Δ0*, *met15Δ0*, *ura3Δ0*) was transformed with 2*μ* expression plasmids encoding N-terminally ZZ-UBL-tagged Citrine [41,42] under control of the *GAL1* promoter (see S2 Table). Single colonies were grown over night in CSM-Ura containing 2% glucose and 2% raffinose. Cells were washed in CSM-Ura + 2% raffinose, diluted to OD_600_ = 0.2 and shaken over night at 30°C. Protein expression was induced by adding 2% galactose for 5 h. Total lysates were prepared by the NaOH/TCA method (modified from [43]) and analyzed by Western blot. First, the ZZ-tag was directly detected using a fluorescently labeled rabbit IgG (anti-mouse) coupled to IRDye680 (LI-COR #926–32220; 1:2500 dilution). After blocking with 0.1 mg/ml human IgG, the membrane was probed with a polyclonal rabbit anti-GFP primary antibody recognizing Citrine and CFP (0.7 μg/ml final concentration) followed by a goat anti-rabbit secondary antibody coupled to IRDye800CW (LI-COR #926–32211; 1:5000 dilution). Blots were scanned with an Odyssey infrared imaging system (LI-COR).

For protein purifications from yeast, cells extracts were prepared by glass bead lysis (modified from [44]) in LS-S buffer containing protease inhibitors (100 μg/ml E-64, 5 mM EDTA, 100 μg/ml chymostatin, 100 μM leupeptin, 10 μM pepstatin A). Cleared lysates were incubated with an anti-ZZ affinity resin. After washing off unbound material, target proteins were eluted with the appropriate protease within 1 h at 4°C. Material remaining on the resin was analyzed after elution with SDS sample buffer.

## Results

Recently, we found that the *S*. *cerevisiae* Atg4 protease (scAtg4) can efficiently cut off N-terminal tags from recombinant scAtg8 fusion proteins. Thereby, scAtg4 displays orthogonal specificity to the excellent tag-cleaving proteases bdSENP1 and bdNEDP1 [33]. Yet, the system suffered from low solubility of the scAtg4 protease following bacterial expression, limited salt tolerance of the cleavage reaction as well as from low expression levels of scAtg8 fusions. We therefore aimed at overcoming these shortcomings while preserving the excellent specificity profile of the scAtg8/scAtg4 system and its good cleavage efficiency at low temperature. To this end, we searched for Atg4 and Atg8 orthologs in eukaryotes with low temperature optimum. Based on these criteria, we decided to further characterize the Atg4B protease from *Xenopus laevis* (xAtg4B) and its Atg8 substrates xLC3B and xGATE16 (sequence alignments with the respective human orthologs are shown in S1 Fig).

### Effect of xLC3B on the expression level and solubility of target proteins

It is established that certain N-terminal tags can enhance expression levels and solubility of recombinant proteins (for review see e.g. [45,46]). To elucidate how an xLC3B tagging module would perform in this respect, we fused various PolyHis-UBL modules to the N-terminus of GFP and compared the expression of the resulting fusions in *E*. *coli* (S2 Fig). xLC3B-GFP could indeed be highly over-expressed and yielded nearly 3-times more soluble GFP than the corresponding scAtg8 fusion. The highest expression levels were obtained when fusing the recently described bdNEDD8 [33] to GFP. With regard to expression levels, both xLC3B and bdNEDD8 clearly outperformed scSUMO, which is already well known for its expression- and solubility-enhancing effects [47,48].

### Design, expression and purification of xAtg4B fragments

The structures of the free human Atg4B [49,50] and LC3B-bound Atg4B [51] revealed that this cysteine protease combines a papain-like domain with another unique domain that contributes to substrate recognition. Additional contacts are formed within a groove on the protease surface, which accommodates the flexible C-terminus of Atg8-like substrates and thus directs the substrate's C-terminal Gly residue to the active site. The protease's flexible N-terminus may fold back onto the substrate-binding groove and has been implicated in a negative regulation of substrate interactions [51]. Human Atg4B contains also a C-terminal extension, which had to be deleted before substrate-bound Atg4B would crystallize [51]. In the so far available substrate-free structures [49,50], this C-terminal extension was poorly resolved and folded back on the substrate interaction surface, which might suggest that it interferes with substrate binding. Thus, it was tempting to assume that the N- and C-terminal extensions would have an auto-inhibitory effect and that their deletions could boost the proteolytic activity.

**Fig 1:**
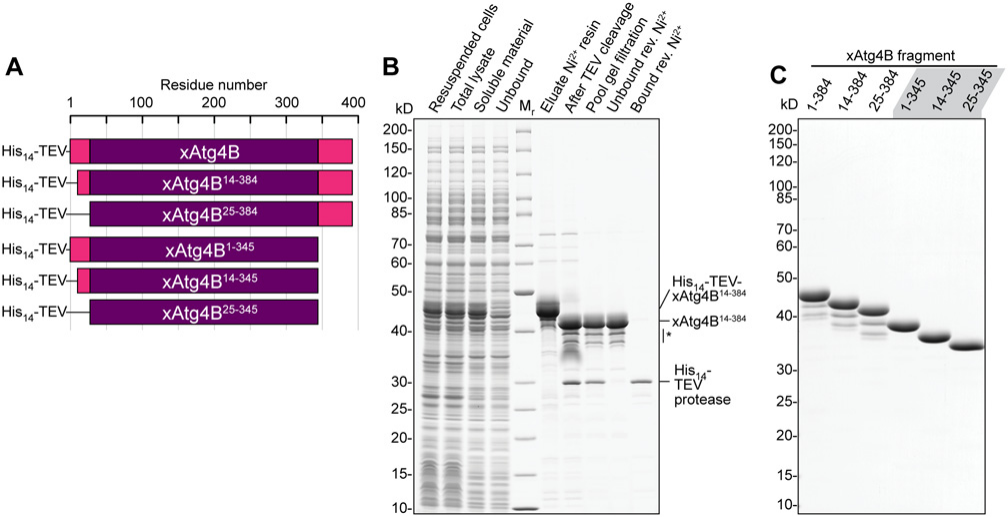
Bacterial expression and purification of xAtg4B protease fragments. **A**, Schematic illustration of expression constructs used for (**B**) and (**C**). **B**, Exemplary purification of xAtg4B^14-384^. His_14_-TEV-xAtg4B^14-384^ was over-expressed in *E*. *coli* strain NEB Express. After cell lysis and centrifugation, the soluble material was applied to a Ni^2+^ chelate resin. Bound proteins were eluted with imidazole and treated with polyHis-tagged TEV protease over night at 4°C before loading on a Superdex 200 gel filtration column. The pooled peak fractions mainly containing cleaved xAtg4B^14-384^ and TEV protease were subjected to a reverse Ni^2+^ chromatography step (rev. Ni^2+^). Here, the polyHis-tagged TEV protease bound to the resin while pure xAtg4B^14-384^ was found in the unbound fraction. Purification of other xAtg4B fragments was done identically. Minor amounts of degradation bands (*) originate from cleavage within the flexible C-terminus. **C**, Purity of xAtg4B protease fragments. 40 pmol (≈1.6 μg) of purified protease fragments were separated by SDS-PAGE and stained with Coomassie G250.

We therefore cloned and expressed not only a full-length version of xAtg4B (residues 1–384), but also five shorter xAtg4B fragments with truncated N- and/or C-termini (Fig 1). All proteases variants could be highly over-expressed in *E*. *coli*. After an initial Ni^2+^-chelate chromatography step, their N-terminal His-TEV tags were cut off by His-tagged TEV protease. The resulting mixture was subjected to gel filtration, after which highly pure tag-free proteases were recovered as non-bound fractions in a reverse Ni^2+^ chelate step. The overall yield was typically >120 mg per liter culture, which is 10–20 times more than obtained for the yeast ortholog scAtg4 (typically 5–10 mg).

### Activity of xAtg4B fragments on xLC3B and xGATE16 fusion proteins

**Fig 2:**
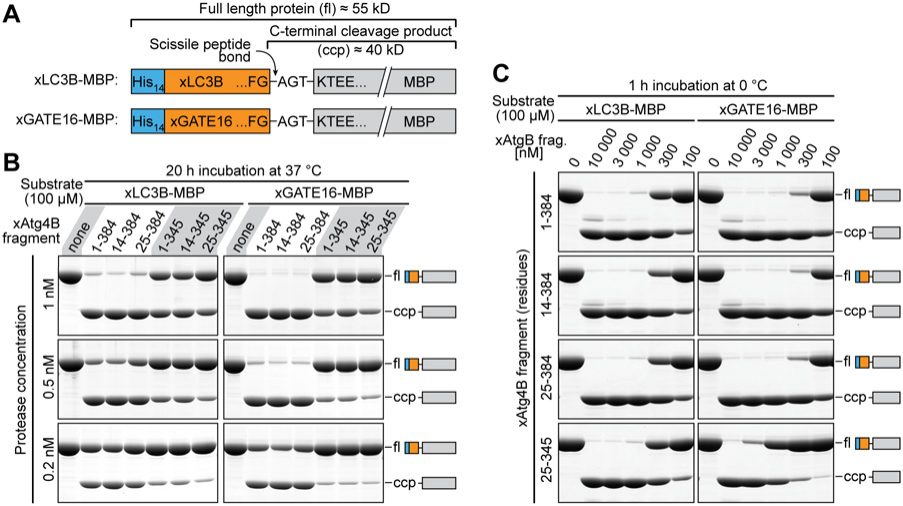
*In-vitro* substrate cleavage by xAtg4B fragments. **A**, Schematic representation of the protease substrates xLC3B-MBP (top) and xGATE16-MBP (bottom). Both fusion proteins contain an N-terminal polyHis-tag, a protease recognition site (xLC3B or xGATE16) and MBP (*E*. *coli* maltose-binding protein, MBP) as a model target protein. To ensure a comparable accessibility, the scissile bond is followed by the identical tri-peptide (AGT; Ala-Gly-Thr) in both substrate proteins. For simplicity, substrate names do not contain the polyHis-tag. **B**, *In-vitro* cleavage assay. 100 μM substrate (xLC3B-MBP (left) or xGATE16-MBP (right)) was incubated for 20 h at 37°C with defined concentrations of the indicated xAtg4B protease fragments. Cleavage products were separated by SDS-PAGE and stained with Coomassie G250. Shown are full-length substrate proteins (fl) and the C-terminal cleavage products (ccp). To estimate the completeness of cleavage, the band intensities were compared to a cleavage standard (see S3 Fig). **C**, Activity of xAtg4B fragments at 0°C. Indicated concentrations of xAtg4B protease fragments were incubated for 1 h at 0°C with 100 μM of the substrates sketched in **A.** For a similar comparison of xAtg4B fragments at 25°C see S4 Fig For examples of complete gels see S5 Fig.

The full-length xAtg4B protease and the five fragments were then analyzed by *in vitro* cleavage assays [33] using two analogous substrate proteins with different protease recognition sites, namely xLC3B or xGATE16, respectively (Fig 2A). For a direct comparison, all reactions of a given experimental setup were performed in parallel for all analyzed protease variants and substrates (see Supporting information for the full set of experiments). For quantification, we used a series of standards, in which non-digested and fully digested substrate had been pre-mixed at defined ratios (S3 Fig).

In a first set of experiments, we incubated 100 μM substrate for 20 hours at 37°C with a concentration series of each protease version. As seen from Fig 2B, 1 nM of the full length protease was sufficient for a near complete cleavage of the xLC3 substrate, which implies that each protease molecule cleaved, on average, ≈100 000 substrate molecules during the incubation. The deletion of the first 13 residues in the xAtg4B^14-384^ variant resulted in a small (≈20%), but reproducible, increase in product formation. This small increase was lost when the deletion was extended to the first 24 residues (xAtg4B^25-384^ variant). Unexpectedly, the deletion of the C-terminal extension had a deleterious effect and decreased the rate of product formation by a factor of ≈ 4. Using the xGATE16 substrate gave similar results, differences being that it was cleaved ≈2-fold faster by the protease version with intact C-terminus and that the deletion of the C-terminal extension caused an even ≥10-fold drop in activity.

To best preserve the activity of target proteins, an ideal tag-cleaving protease should be able to cleave substrates quickly and at low temperatures (0–4°C). We therefore also compared the concentrations of selected xAtg4B fragments protease required for complete substrate cleavage in cleavage reactions performed for 1 h on ice (Fig 2C). Also in this assay, xAtg4B^14-384^ performed best. Less than 1 μM xAtg4B^14-384^ was needed for near complete cleavage of 100 μM xLC3B-MBP within one hour at 0°C. In the case of the xGATE16-MBP even less protease (0.3 μM) was sufficient. Thus, even on ice, xAtg4B^14-384^ can process a several hundred-fold excess of substrate per hour. Strikingly, at 0°C the C-terminal protease deletion did not significantly affect cleavage of xLC3B-MBP while xGATE16-MBP processing was strongly impaired.

### Impact of N- and C-terminal xAtg4B truncations on substrate interaction

**Fig 3:**
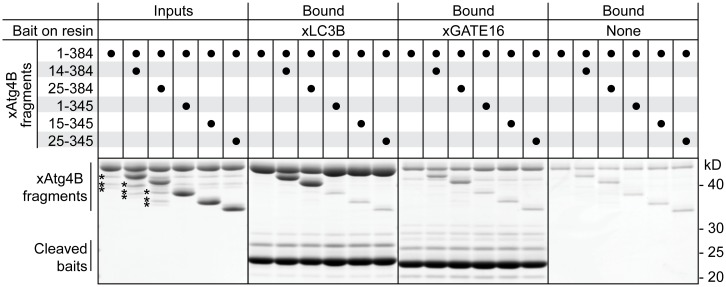
Competitive binding of xAtg4B fragments to immobilized xLC3B and xGATE16. An equimolar mixture of full-length xAtg4B and indicated fragments (10 μM each) was incubated with immobilized xLC3B or xGATE16. A resin without bait protein (right panel) served as a specificity control. Bound proteins were analyzed by SDS-PAGE. xAtg4B degradation products lacking parts of the C-terminal extension are marked with an asterisk (*) in the input fractions. Note that binding is markedly reduced for protease fragments harboring C-terminal deletions. The pull-down efficiency is generally higher when using xLC3B instead of xGATE16 as bait. For complete SDS-PAGE gels see S6 Fig.

At this point, it was unclear how the deletion of the protease's C-terminus slowed down the substrate turnover. We therefore addressed the impact of the N- and C-terminal protease extensions on the most likely parameters (i.e. substrate binding, substrate processing and protease stability) separately. First, we directly compared binding of N- and/or C-terminally shortened protease fragments with the full-length enzyme by competitive pull-down assays using equimolar binary protease mixtures as a prey (Fig 3). In this setup, even small differences in affinity should affect the relative protease stoichiometries between the input and the bound fractions. Immobilized xLC3B pulled down full-length xAtg4B and the N-terminally shortened fragments with the same efficiency. The N-terminal protease truncations hence did not influence binding. In contrast, the interaction of all protease fragments lacking the C-terminal extension was reduced to background levels in the presence of competing full-length protease. Interestingly, also degradation products of the protease lacking less than 39 residues from the C-terminus (unintentionally present in the enzyme preparations) bound xLC3B far less efficiently than the respective enzymes with full-length C-termini, showing that even the extreme C-terminus significantly contributes to substrate binding. Similar results were obtained when using xGATE16 as a bait. The interaction of all proteases with xGATE16 was, however, significantly weaker than with xLC3B.

Thus, xAtg4B's C-terminal extension substantially contributes to recognition of both xLC3B and xGATE16 and is therefore required for robust substrate cleavage. In contrast, the first N-terminal residues of the protease do not affect substrate binding.

### Concentration dependence of substrate processing

The concentration of protease needed for complete substrate processing will not only depend on the temperature and incubation time, but also on the concentration of the substrate and the eventually formed (inhibitory) end-product. To address this issue, we analyzed substrate cleavage at a constant protease/substrate ratio, while varying the concentrations of both, substrate and protease, proportionally.

**Fig 4:**
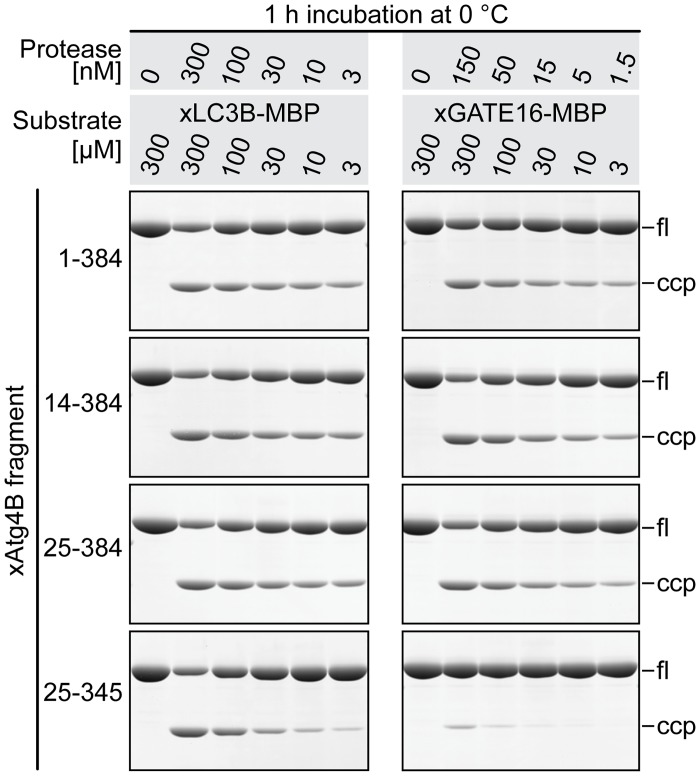
Cleavage efficiency at limiting substrate concentrations. The concentration of indicated protease fragments and the substrates xLC3B-MBP (left) or xGATE16-MBP (right) was titrated at constant protease/substrate ratio (1:1000 or 1:2000, respectively). After cleavage (1 h at 0°C), a fraction of each reaction corresponding to 1.2 μg (≈20 pmol) of substrate protein was analyzed by SDS-PAGE. Due to the different substrate concentrations, the absolute volume of the cleavage reaction analyzed by SDS-PAGE had to be adjusted accordingly.

At a saturating (300 μM) concentration of xLC3B substrate, all analyzed protease variants were similarly active, clearly showing that the C-terminus of xAtg4B is dispensable for the actual catalytic step (Fig 4, left). At higher dilutions, however, clear differences became apparent: While full-length xAtg4B and both N-terminally shortened fragments cleaved the xLC3B substrate rather efficiently even at substrate concentrations as low as 3 μM, the C-terminally truncated protease showed significantly reduced cleavage already at 30–100 μM substrate concentration (Fig 4, left lower panel).

The processing of the xGATE16 substrate was, in this experimental setup, more sensitive to dilution than the xLC3 substrate (Fig 4, right). This is consistent with its weaker binding to the protease (Fig 3). At substrate saturation, however, the xGATE16 fusion required less protease for efficient processing. Such faster cleavage cycles can be explained by a faster release of (the more weakly binding) xGATE16 cleaving products from the enzyme. The deletion of the protease's C-terminus impaired xGATE16 processing so strongly that not even the increase of the substrate concentration to 300 μM could compensate for the weakened interaction (Fig 4, right lower panel).

### Thermal stability of xAtg4B protease fragments

**Fig 5:**
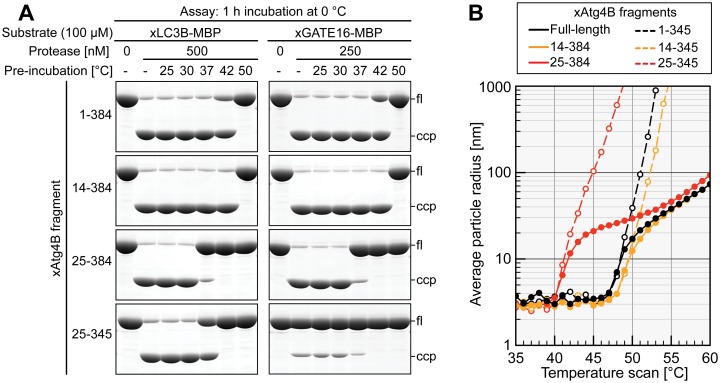
Thermal stability of xAtg4B fragments. **A,** Long-term thermal stability. Indicated xAtg4B fragments were pre-incubated for 16 h at indicated temperatures in the presence of 20 mM DTT under argon to protect the active site cysteines from oxidation. The remaining activity was then assayed by treating 100 μM of xLC3B-MBP or xGATE16-MBP substrate with each protease fragment for 1 h at 0°C. **B,** Dynamic light scattering (DLS) analysis. xAtg4B fragments were diluted to a final concentration of 10 μM and assayed by DLS. The temperature was automatically raised by 1°C every 10 min. DLS signals were acquired just before each temperature step.

We also wanted to explore in how far the above described protease variants differ in respect of their thermal stability. To this end, we pre-incubated each of xAtg4B variants for 16 h at 25, 30, 37, 42, or 50°C before analyzing their remaining activity in a standard xLC3B cleavage assay at 0°C (Fig 5A, left). In this assay, the full-length enzyme retained full activity after over-night incubation at 37°C, but lost activity at higher temperatures. xAtg4B^14-384^ turned out to be the most thermostable variant; it survived at least 42°C for 16 h. A drastic loss in activity was, however, observed already at 37°C for enzyme fragments lacking the N-terminal 24 residues. Identical effects were obtained when using the xGATE16 cleavage assay as a readout (Fig 5A, right).

In a second assay, we used dynamic light scattering (DLS) to analyze the thermal denaturation of our xAtg4B fragments (Fig 5B). The full-length enzyme started to unfold at 47–48°C. Fragments lacking the N-terminal 13 residues were slightly stabilized (by 1–2°C) while an N-terminal deletion of 24 residues reduced the temperature stability by 7–8°C. All tested enzymes with an intact C-terminus showed biphasic denaturation curves, pointing to distinct steps of initial unfolding and subsequent aggregation. A deletion of the C-terminal extension did not significantly change the onset of denaturation (Fig 5B, compare solid with dashed lines), but promoted subsequent aggregate formation. The strongly negatively charged C-terminus might thus act as a solubility enhancer preventing immediate aggregation.

Interestingly, the temperatures required to observe an initial decline of enzymatic activity (Fig 5A) were generally ≈5°C lower than the onset of thermal denaturation observed by DLS (Fig 5B). This discrepancy could be resolved by a long-term DLS experiment with xAtg4B^25-384^ at 37°C (S7 Fig): Here, during the initial two hours of incubation, the protease appeared rather stable. At longer incubation, however, xAtg4B^25-384^ started to unfold and aggregate. The discrepancy between the activity assay (after 16 h of thermal denaturation) and the DLS experiment (temperature increase 1°C per 10 min) can thus most likely be explained by the different experimental time-scales.

### Characterization of the optimal xAtg4B^14-384^ fragment

The described assays so far show that xAtg4B^14-384^ combines optimal stability and substrate processing. We therefore focused on this optimal fragment to analyze its application for removal of tags from recombinant proteins.

#### Time course

**Fig 6:**
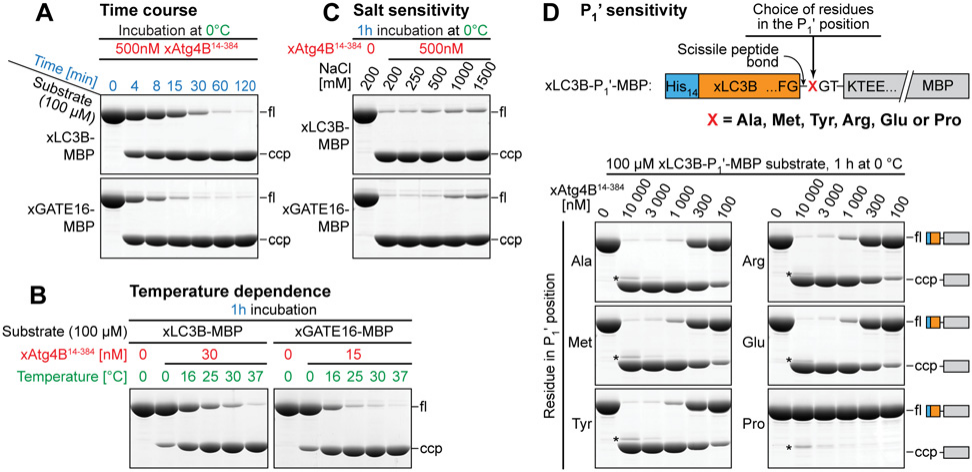
*In-vitro* cleavage characteristics of xAtg4B^14-384^. **A,** Time course. Substrates (100 μM) were incubated at 0°C with 500 nM of xAtg4B^14-384^. At indicated time points, aliquots were withdrawn. Cleavage products were separated by SDS-PAGE and stained with Coomassie G250. Shown are full-length substrate proteins (fl) and the C-terminal cleavage products (ccp). For a side-by side comparison of selected protease fragments see S4 Fig. **B**, Temperature dependence of substrate cleavage. 100 μM of xLC3B-MBP (left) or xGATE16-MBP (right) were incubated with xAtg4B^14-384^ for 1 h at defined temperatures. Note that in comparison to the xGATE16-MBP substrate, twice as much protease was used for cleavage of the xLC3B-MBP substrate. For a comparison of selected protease fragments see S8 Fig. **C,** Salt sensitivity. 100μM of each substrate was incubated for one hour at 0°C with 500 nM xAtg4B^14-384^ at NaCl concentrations ranging from 0.2 to 1.5 M. For a comparison of selected protease fragments see S8 Fig. **D,** P_1_' preference. Protease substrates used to analyze the P_1_' preference of xAtg4B^14-384^ followed the general outline shown in Fig 2A. Here, however, the P_1_' position of the P_1_-P_1_' scissile bond had been mutated to the potentially non-preferred residues methionine (Met), tyrosine (Tyr), arginine (Arg), glutamic acid (Glu), or proline (Pro). Solution cleavage assays were performed with indicated concentrations of xAtg4B^14-384^ for 1 h at 0°C. Bands marked with an asterisk (*) refer to the protease.

We first performed a time course at 0°C with 0.5 μM protease and 100 μM substrate to determine the minimal time required for substrate cleavage (Fig 6A). Fully consistent with earlier results (Fig 2C), near complete cleavage of the xLC3B substrate occurred within 60 minutes. Cleavage of the xGATE16 substrate was even ≈4-fold faster, indicating that complete substrate cleavage is possible within a very short time frame using low protease concentrations and mild cleavage conditions.

#### Temperature dependence

Next, we analyzed the temperature dependence of substrate processing by the optimal xAtg4B^14-384^ protease fragment (Fig 6B). We observed a remarkable activity boost when the incubation temperature was increased from 0°C to 16°C or 25°C. At 25°C for example, the enzyme was able to process within one hour a ≈7000-fold excess of the xGATE16 substrate to completion. Such higher incubation temperature is certainly an option when the target protein is not particularly sensitive to denaturation.

#### Salt sensitivity

When purifying proteins, one should ideally have the freedom to choose the composition of used buffers such that stability and functionality of the target proteins are best preserved. The activity profile of a used tag-cleaving protease should then impose as little as possible constraints. Systematic tests (Fig 6C) now revealed that the xAtg4B protease fragment is highly active not only at moderate salt (200 mM NaCl), but also at very high salt concentrations such as 1 M or 1.5 M NaCl. This contrasts the yeast homolog scAtg4, which showed a considerable activity loss already at 1 M NaCl [33].

#### P1' sensitivity of xAtg4B14-384

If target proteins with a defined (e.g. the authentic) N-terminus are to be produced, the enzyme's sensitivity to the residue in the P_1_' position (i.e. the residue following the scissile bond; Fig 6D) becomes an important parameter. An optimal enzyme will offer maximum freedom to choose any desired residue in the P_1_' position. We therefore analyzed the protease concentration required for cleavage of several analogous substrates with altered residues in the P_1_' position (Fig 6D). Surprisingly, the enzyme showed remarkable promiscuity and required only slightly more protease for efficient cleavage of substrates harboring Met, Tyr, Arg or Glu in the P_1_' position as compared to the original P_1_'_Ala_ substrate. The enzyme, however, was unable to process a P_1_'_Pro_ substrate.

### One-step protein purification of target proteins expressed in *E*. *coli* by on-column cleavage using xAtg4B^14-384^

**Fig 7:**
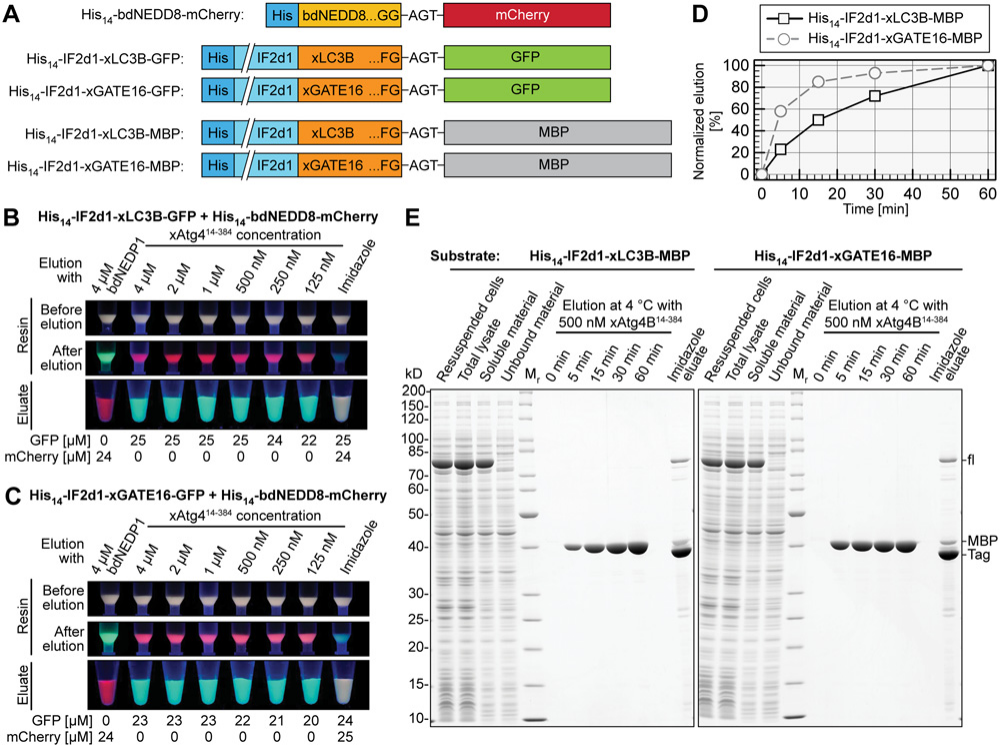
On-column cleavage using xAtg4B^14-384^. **A**, Schematic representation of substrate proteins used in (**B**)—(**E**). The N-terminal domain of *E*. *coli* IF2 (IF2d1 [65,66]) serves as a spacer. **B and C**, A silica-based Ni^2+^ chelate resin was pre-loaded with similar amounts of His_14_-bdNEDD8-mCherry and either His_14_-IF2d1-xLC3B-GFP (**B**) or His_14_-IF2d1-xGATE16-GFP (**C**). 50 μl aliquots were treated with indicated concentrations xAtg4B^14-384^ for 1 h at 4°C. Control incubations were performed with 4 μM bdNEDP1 or with buffer containing 400 mM imidazole. Resins and eluates were photographed while illuminated at 366 nm. GFP and mCherry in the eluate fractions were quantified via their specific absorptions. Quantification results are given below the respective eluate fractions. **D and E**, Protein purification using on-column cleavage by xAtg4B^14-384^. Indicated substrates were over-expressed in *E*. *coli*. After lysis and ultracentrifugation, the soluble material was incubated with a Ni^2+^ chelate resin. The resin was washed and treated with 500 nM xAtg4B^14-384^ at 4°C. At indicated time points, the concentration and purity of the released MBP was determined using the calculated absorption coefficient at 280 nm (OD_280_) and SDS-PAGE, respectively. Proteins remaining on the resin after 60 min were eluted by 500 mM imidazole. The time course of elution is shown in (**D**), the OD_280_ reading at 60 min elution time was set to 100%. Relevant steps of the purifications are shown in (**E**).

An important application of tag-cleaving proteases is on-column cleavage of recombinant proteins. We directly addressed the suitability of xAtg4B^14-384^ for this purpose using polyHis-tagged substrate proteins bound to a silica-based Ni^2+^ chelate resin of high porosity (Fig 7). More specifically, ≈100 μM of His_14_-IF2d1-xLC3B-GFP or His_14_-IF2d1-xGATE16-GFP were immobilized on the respective matrices along with the control protein His_14_-bdNEDD8-mCherry (Fig 7A) before incubation with defined concentrations of xAtg4B^14-384^ or the bdNEDD8-specific protease bdNEDP1 for 1 h at 4°C. Under these conditions, 250–500 nM of xAtg4B^14-384^ was sufficient for near-quantitative elution of GFP from the silica-based resin (Figs 7B and 7C). The cleavage was exceedingly specific as even at much higher concentrations of xAtg4B^14-384^ no elution of the bdNEDD8-tagged mCherry control protein could be detected. Vice versa, after treatment with a high concentration of bdNEDP1, only mCherry but no GFP could be detected in the eluates. When using a Sepharose-based resin with high porosity, only slightly higher protease concentrations were required for efficient elution (not shown). The elution efficiency was, however, significantly reduced when matrices with low porosity or substrate proteins without flexible linker between the polyHis tag and the protease recognition site were used (data not shown).

We exploited the xAtg4B/xLC3B protease/substrate pair to purify the model target protein maltose-binding protein (MBP) by on-column cleavage of either His_14_-IF2d1-xLC3B-MBP or His_14_-IF2d1-xGATE16-MBP (Figs 7D and 7E). Even at moderate induction both proteins were highly over-expressed in *E*. *coli* and displayed excellent solubility (Fig 7E). Stronger induction led to massive over-expression of fusion proteins without compromising their solubility (not shown). About 160–200 μM of each fusion protein was immobilized on a Ni^2+^ chelate resin and treated with 500 nM xAtg4B^14-384^ at 4°C. Strikingly, the initial cleavage rate was very high when using the xGATE16 fusion protein (Fig 7D). Here, >80% and >90% of the MBP target protein was released already after 15 min and 30 min, respectively. At the corresponding points in time, the xLC3B fusion protein was processed to only ≈50% and 75%. In both cases, however, efficient release of highly pure MBP was reached within one hour (Figs 7D and 7E).

### Cross-reactivity with other tag-cleaving proteases

**Fig 8:**
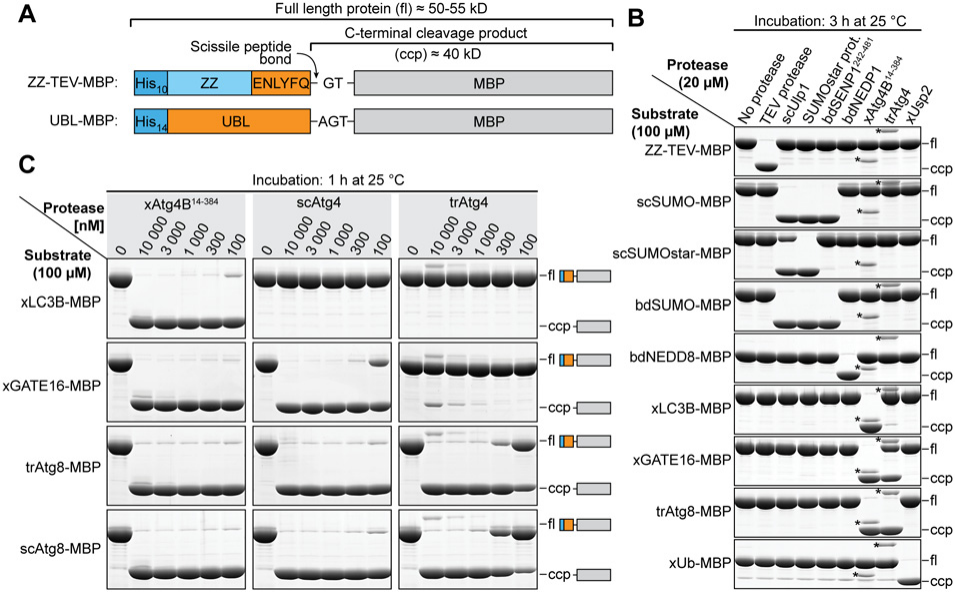
*In-vitro* cross-reactivity with other tag-cleaving proteases. **A**, Schematic representation of substrates used for (B) and (C). The TEV protease substrate contains an N-terminal His_10_-ZZ tag preceding the TEV protease recognition site. All other substrates follow the scheme described in Fig 2A, the protease recognition site, however, is replaced by the respective ubiquitin-like protein (UBL). **B**, Cross-reactivity between recombinant tag-cleaving proteases. bd, Brachypodium distachyon; tr, *Triticum aestivum* (summer wheat); xUb, *Xenopus* ubiquitin. Bands marked with an asterisk (*) originate from the respective protease. For complete gels see S6 Fig. **C**, Detailed titration analysis of cross-reactivity between *Xenopus laevis* (x), *S*. *cerevisiae* (sc) and wheat (tr) Atg4 homologs.

A crucial parameter for the practical application of tag-cleaving proteases is their substrate specificity. This parameter is especially important when mutually exclusive specificity ("orthogonality") to other proteases is strictly required, e.g. for purification of protein complexes with controlled subunit stoichiometry [34]. Also, it is important to know which host proteases could potentially cleave a given protease recognition site during expression. For practical applications, we were especially interested in the cross-reactivity of xAtg4B with the well-established TEV protease [52,53], scUlp1 [32], SUMOstar protease [35,36] and the recently described proteases bdSENP1, bdNEDP1, and xUsp2 [33,34]. In addition, we also included the wheat (*Triticum*) Atg4 ortholog (trAtg4). In order to analyze the specificity profiles of these proteases, we incubated a high concentration (20 μM) of each protease with 100 μM of each substrate protein (see Fig 8A) in all possible combinations for 3 h at 25°C (Fig 8B). For all proteases except TEV protease, these conditions correspond to a significant (>200- to 30 000-fold) over-digestion. Under these conditions, both xAtg4B^14-384^ and trAtg4 only cleaved substrates containing Atg8-like UBLs (xLC3B, xGATE16 or trAtg8), but none of the substrates dedicated to other proteases. Vice versa, substrates containing Atg8-like UBLs were exclusively cleaved by Atg4 proteases. Atg4 proteases and Atg8-type substrate proteins are therefore truly orthogonal to all other protease/substrate pairs analyzed. Within the Atg8-type substrates, interesting differences became apparent: While xLC3B was nearly exclusively recognized by xAtg4B^14-384^, both xGATE16 and trAtg8-containing substrates were in addition also cleaved by trAtg4.

We further analyzed these inter- and intra-species substrate preferences of Atg4-like enzymes using detailed protease titration assays (Fig 8C). Here, we also included the *S*. *cerevisiae* Atg4 ortholog (scAtg4) along with its cognate substrate scAtg8 that were described recently [33]. In this assay, xAtg4B showed the broadest substrate promiscuity and cleaved a 1000-fold excess of all four substrate proteins containing xLC3B, xGATE16, trAtg8 or scAtg8 within 1 h at 25°C (Fig 8C, left column). The yeast scAtg4 protease efficiently processed xGATE16, trAtg8 and scAtg8, but was completely unable to cleave the xLC3B substrate (Fig 8C, middle column). The *Triticum* protease trAtg4 cleaved only its cognate substrate trAtg8 and the yeast substrate with decent efficiency (Fig 8C, right column). In comparison, the *Xenopus* xGATE16 substrate required drastically (>100-fold) higher trAtg4 concentrations for significant cleavage; xLC3B cleavage by trAtg4 was only barely detectable.

### Stability of xLC3B and xGATE16 fusion proteins in eukaryotic systems

**Fig 9:**
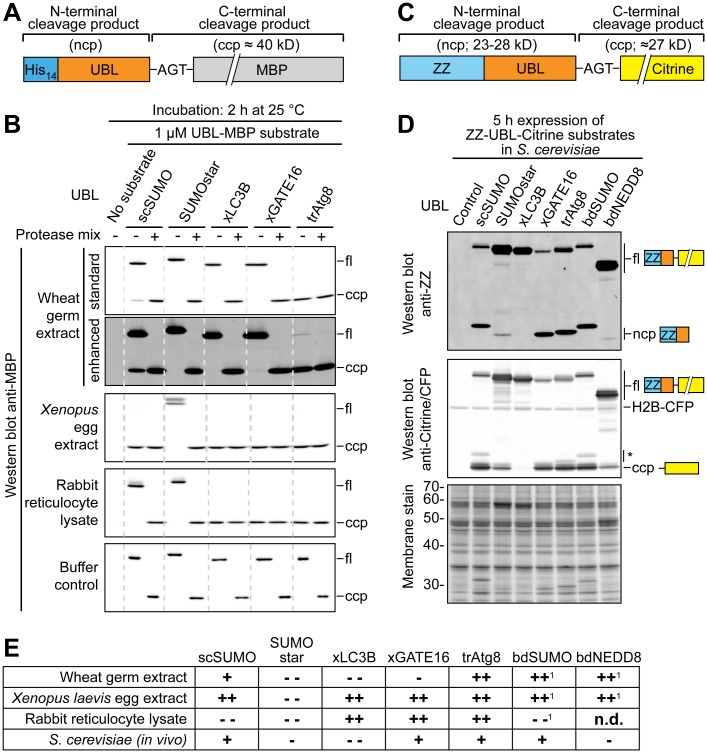
Stability of UBL fusions in eukaryotic lysates and in *S*. *cerevisiae*. **A**, Schematic representation of substrates used for (**B**). **B**, Stability of protease substrates in cell extracts. Note that in wheat germ extract no proteolytic fragments originating from SUMOstar-, xLC3B- or xGATE16-containing substrates can be detected. For complete blots and stained membranes see S10 Fig. **C**, Schematic representation of substrates used for expression in *S*. *cerevisiae* (**D**) harboring an N-terminal ZZ-tag, an ubiquitin-like protein (UBL) and a C-terminal Citrine. **D**, *In-vivo* stability of protease substrates in *S*. *cerevisiae*. Indicated protease substrates were over-expressed in a *S*. *cerevisiae* strain constitutively expressing H2B-CFP. Total cell lysates were analyzed by Western blot with antibodies recognizing the ZZ-tag (upper panel) or Citrine and CFP (middle panel). Equal loading was confirmed by staining the membrane after blotting (lower panel). Bands marked with an asterisk (*) originate from ZZ-tagged proteins cross-reacting with the anti-Citrine/CFP antibody. For complete original blots and stained membranes see S11 Fig. **E**, Cleavage of UBL substrates in extracts and in *S*. *cerevisiae*. ++, highly efficient cleavage; +, cleavage;–, traces cleaved;––, no cleavage; n.d.: not determined; ^1^, data not shown.

The unexpectedly high resistance of xLC3B towards cleavage by Atg4-like proteases originating from other species encouraged us to address the stability of xLC3B and xGATE16 fusions in various eukaryotic cell extracts (Figs 9A and 9B). For control purposes, we also included analogous fusions to trAtg8, scSUMO and the cleavage-resistant scSUMO variant SUMOstar [35,36]. As expected, in wheat germ extract 1 μM of xLC3B- or xGATE16-containing substrate proteins were not significantly processed within 2 h at 25°C, while the corresponding trAtg8 fusion was completely cleaved. In comparison, all substrate proteins harboring Atg8 homologs were completely cleaved both in *Xenopus* egg extract and rabbit reticulocyte lysate. Interestingly, the scSUMO fusion was only partially cleaved in wheat germ extract and remained stable in rabbit reticulocyte lysate. Control incubations containing a protease mix (0.1 μM each of scUlp1, SUMOstar protease, xAtg4B^14-384^ and trAtg4) confirmed that the extracts did not contain any substances inhibiting specific proteolytic substrate processing.

Next, we wanted to find out if some of the analyzed UBL-like protease recognition sites are also compatible with production of intact full-length recombinant fusion proteins in a living eukaryotic host. We therefore over-expressed various ZZ-UBL-Citrine fusion proteins (Fig 9C) in *S*. *cerevisiae* under the control of the *GAL1* promoter. In line with the *in-vitro* cleavage experiments presented before (Fig 8C), even after 5 h of induction, the xLC3B fusion remained completely intact. In contrast, the scSUMO-, xGATE16-, trAtg8- and bdSUMO-fusions were largely cleaved by endogenous yeast proteases. Much to our surprise, also the "cleavage-resistant" SUMOstar variant [35,36] was not completely inert *in vivo* as both, N-terminal and C-terminal cleavage products could be detected using specific antibodies (Fig 9D). Unexpectedly we found that a fusion protein containing bdNEDD8 was even more resistant towards *in vivo* cleavage than the SUMOstar substrate. These findings suggest that xLC3B and the previously introduced bdNEDD8 [33] could potentially be used as protease recognition sites for the recombinant expression of intact full-length fusion proteins in *S*. *cerevisiae*.

### One-step purification of proteins expressed in *S*. *cerevisiae*

**Fig 10:**
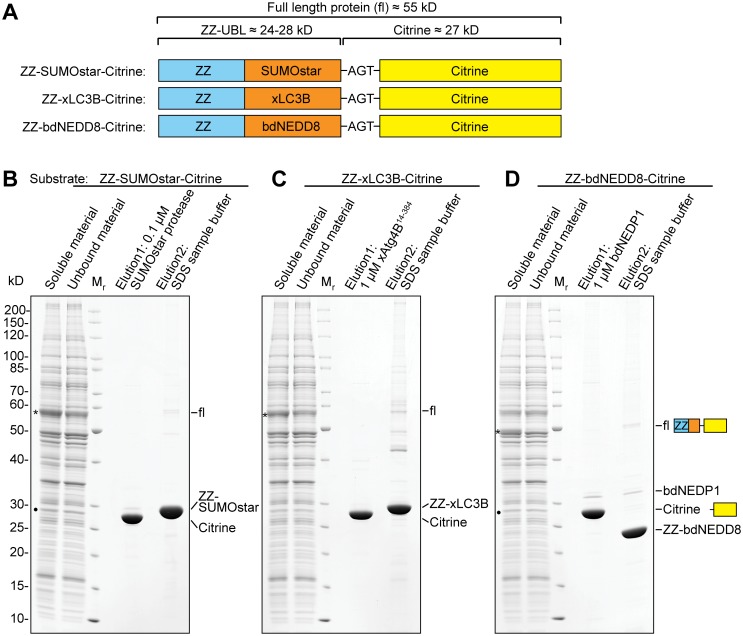
One-step protein purification from *S*. *cerevisiae*. ZZ-UBL-Citrine fusions sketched in (**A**) were over-expressed in *S*. *cerevisiae*. Cells were lysed and the soluble material was incubated with an anti-ZZ affinity resin. After washing off unbound material, highly pure Citrine was eluted by treatment with 0.1 μM SUMOstar protease (**B**), 1 μM xAtg4B^14-384^ (**C**) or 1 μM bdNEDD8 (**D**) for 1 h at 4°C. Material remaining on the resin was analyzed after elution with SDS sample buffer. The asterisk (*) denotes the full-length xLC3B fusion protein. The filled circle (•) marks band partially corresponding to low levels of free Citrine originating from *in-vivo* cleavage of the respective SUMOstar and bdNEDD8 fusion proteins.

In order to show that the xLC3B/xAtg4B and bdNEDD8/bdNEDP1 systems are indeed suited for purification of recombinant proteins from a eukaryotic host, we purified recombinant Citrine as a model target protein from *S*. *cerevisiae*. To this end, the ZZ-UBL-Citrine fusions were over-expressed in yeast for 5 h as before. After cell lysis in a native buffer, the full-length fusion protein was found in the soluble fraction from which highly pure recombinant Citrine could be obtained by an efficient one-step affinity capture and on-column cleavage procedure (Fig 10).

## Discussion

Tag-removing proteases are powerful tools in protein biochemistry. Although several proteases are routinely used for this purpose [31,32,45–47,54], most of them have severe drawbacks including low specific activity, limited specificity or strict constraints concerning temperature, buffer requirements or sequence context. Recent work from our lab has introduced bdSENP1 and bdNEDP1, two new proteases that are largely devoid of these limitations [33]. More elaborate applications, however, may require multiple proteases with optimal features at the same time [34]. We thus aimed at finding new proteases that could potentially be used for tag removal. To this end, we characterized the *Xenopus laevis* xAtg4B protease along with two of its substrates, xLC3B and xGATE16. More specifically, we were interested in finding well-behaved and stable protease fragments with optimal proteolytic activity.

### Significance of N- and C-terminal xAtg4B extensions

Based on the known structure of the human Atg4B ortholog (hsAtg4B) [49–51], we designed a series of xAtg4B fragments with N-terminal and C-terminal truncations. At low temperature, the analyzed N-terminally truncated xAtg4B fragments (xAtg4B^14-384^ and xAtg4B^25-384^) showed a catalytic activity comparable to the full-length enzyme. While these results seem to be in contrast to earlier studies on the human Atg4B ortholog that suggested an auto-inhibitory function of the N-terminal extension [27,51], we observed that at temperatures ≥16°C, the two shorter fragments were indeed slightly more active than the full-length enzyme (see S8 Fig). This temperature effect could potentially be a result of several hydrophobic interactions that are observed between the N-terminal extension of the human enzyme and the protease surface near the catalytic center [50]. Importantly, deletion of only 13 N-terminal residues was sufficient to overcome this effect. In addition, this deletion created an enzyme fragment (xAtg4B^14-384^) with superior temperature stability compared to xAtg4B^25-384^. It should, however, be noted that—at least for xAtg4B from *Xenopus laevis*—the effects seen upon shortening its N-terminal extension are rather subtle and thus insufficient to justify its denomination as an "auto-inhibitory domain".

We also analyzed the contribution of the flexible C-terminal protease extension (residues 346–384) to substrate recognition and processing. While the significance of this region so far had not been directly addressed, we now found compelling evidence that it contributes to an efficient interaction with two dedicated xAtg4B substrate proteins, xLC3B and xGATE16. This finding was surprising for two reasons. First, the available structures of the substrate-free human Atg4B (hsAtg4B) suggest that the protease's C-terminus partially occupies the substrate-binding site [49,50]. It therefore has to be displaced before substrate binding can occur, which may thus hamper formation of the protease•substrate complex. Second, crystals of LC3B-bound hsAtg4B could be obtained only after removal of the C-terminal extension [51], which (i) shows that the C-terminus of xAtg4B is not strictly required for substrate interaction and (ii) could indeed suggest an inhibitory effect on complex formation. In contrast, our results clearly show that the C-terminal extension is an integral part of the protease's substrate interaction surface.

In this respect, it is a noticeable coincidence that the C-terminus of xAtg4B is rich in hydrophobic amino acids with Φ-x-x-Φ clusters (where Φ stands for hydrophobic, x for any amino acid) similar to the well-established ARM/LIR motifs (sequence typically Trp-x-x-Leu/Ile) that are characteristic for a variety of Atg8-interacting proteins [55–58]. Although elucidation of the structural details of the interaction between the Atg4B C-terminus and its substrates has to await further investigations that are beyond the scope of this study, it is tempting to speculate that the protease's flexible C-terminus might interact with LC3B and GATE16 in an ARM/LIR-like manner. Such an interaction might in addition be favored by the charge complementarity between the highly acidic C-terminus of Atg4B and a conserved basic surface on LC3B and GATE16. Hydrophobic residues within such ARM/LIR-like motifs could well explain how the protease's C-terminus confers an increased salt tolerance.

The strikingly different affinities of xLC3B and xGATE16 for xAtg4B point towards a model in which xGATE16 processing is largely limited by its binding to the protease, while cleavage of xLC3B substrates is more limited by the rate of the actual catalytic step and/or the product release (at least when substrate concentrations ≥3μM are considered.) As a consequence, the effect of C-terminal protease truncations is only visible when the rate-limiting step is affected: While the C-terminal protease truncation affects processing of xLC3B mainly under stringent conditions (high salt, elevated temperature or low substrate concentration), the effect is pronounced already at standard conditions (0°C, 250 mM NaCl, 100 μM initial substrate concentration) when using the xGATE16 substrate.

In combination, the folded core and the C-terminal extension of xAtg4B mediate a strong interaction with the xLC3B substrate, which is beneficial for efficient substrate processing at high dilution and complete processing of substrates.

### Applications of xAtg4B^14-384^ for protein purification from pro- and eukaryotic hosts

Our best performing xAtg4B fragment, xAtg4B^14-384^, has great potential as a new tag-cleaving protease. This protease fragment is highly active and routinely cleaves a 100- to 200-fold substrate excess within 1 h at 0°C. For comparison, TEV protease, which is probably still the most common tag-cleaving protease, requires 30–50 times higher protease concentrations under these conditions [33]. In addition, xAtg4B^14-384^ shows good temperature stability (≥42°C for 16 h) and can therefore also be used at higher temperatures. At 25°C, e.g., xAtg4B^14-384^ can cleave a 2000-fold substrate excess within one hour; at 37°C even less protease is required for efficient cleavage. When used for *in-vitro* tag removal from recombinant proteins, this high specific activity reduces contamination of the final protein preparation by the protease. Compared to other tag-cleaving proteases like scUlp1 [32,33] or its derivative SUMOstar protease [35], xAtg4B displays a superior salt tolerance (tested up to 1.5 M NaCl) and a broad P_1_' promiscuity, parameters that are important for robust cleavage of recombinant substrate proteins in various buffer conditions and sequence contexts.

When analyzing cross-reactivity with other tag-cleaving proteases, we found out that xAtg4B is fully orthogonal to the recently introduced bdNEDP1 protease as well as to all SUMO-specific proteases (bdSENP1, scUlp1 or its derivative SUMOstar protease). Thus, these three groups of highly efficient proteases ideally complement each other and can be combined to purify protein complexes with controlled subunit stoichiometry by successive affinity capture and proteolytic release steps [34].

Importantly, both analyzed xAtg4B substrates, xLC3B and xGATE16, promote solubility and high-level expression of the respective fusion proteins in *E*. *coli* (see S2 Fig and Fig 7E). This is in striking contrast to their yeast homolog scAtg8, which in direct comparisons consistently produces significantly lower levels of soluble fusion proteins (S2 Fig). All in all, both xAtg4B substrates are promising fusion partners for expression of recombinant target proteins in *E*. *coli* and may at the same time serve as recognition sites for xAtg4B. The best choice between the two possible protease recognition sites might depend on the specific application. While xGATE16 is cleaved more rapidly under standard conditions, xLC3B cleavage is slightly slower but extraordinarily robust.

In addition, xLC3B features remarkable advantages: We found that xLC3B fusions are stable in wheat germ extract and only marginally processed by wheat Atg4 (trAtg4) even under drastic *in vitro* conditions. This suggests that stable xLC3B fusion proteins can also be produced in plants. Furthermore, as demonstrated for the human LC3B ortholog before [22], xLC3B is not recognized by the *S*. *cerevisiae* Atg4 protease either. Stable xLC3B fusions can thus be expressed in a fungal host and purified by a simple one-step capture and proteolytic release strategy. Such eukaryotic expression might be exploited for the production of proteins that rely on the eukaryotic folding machinery or have to be modified by posttranslational modifications. Fully unexpectedly, we found that also bdNEDD8 fusion proteins are only marginally processed in yeast. With xLC3B, bdNEDD8 [33] and SUMOstar [35,36], we thus have three orthogonal UBL-derived protease recognition sites that in principle allow for the production of stable but cleavable fusion proteins in *S*. *cerevisiae* (Figs 9E and 10). Strikingly, amongst these UBLs xLC3B is the only one that is strictly stable *in vivo* while traces of cleavage products originating from the bdNEDD8 substrate and low amounts of cleaved SUMOstar fusions were clearly detected (Figs 9D and 10B). In combination, these UBLs should allow for *in-vivo* co-expression and purification of three-subunit complexes with defined subunit stoichiometry also using yeast as an expression host [34].

It is important to note that xLC3B, xGATE16 or other UBLs including brNEDD8 or brSUMO not only can be used in combination with an N-terminal polyHis-tag. Also, the mentioned UBLs can be combined with virtually any affinity tag [45,46,54,59–63] including, e.g., protein-tags like MBP, GST or the ZZ-tag, peptide tags like the HA-, myc- or FLAG-tags, or an biotinylated Avi-tag. In fact, also fusions to N-terminal non-affinity-tag fusion partners might be advantageous, e.g., for enhancing expression levels or solubility of target proteins, or their detection. Other possible applications include regulated degradation (TIPI system [37,39]) or targeted localization [38]. These techniques have so far mostly been performed using TEV protease. For the TIPI system, however, it has been shown that the poor proteolytic activity and pronounced P_1_' sensitivity of TEV protease is limiting for the proteolytic activation of the degradation signal [64]. Here, xAtg4B with its high activity and pronounced P_1_' promiscuity could potentially have clear advantages over TEV protease.

## Supporting Information

S1 Fig Alignment of human and *Xenopus laevis* Atg4, LC3 and GATE16 orthologs. **A**, Phylogenetic tree of human (hs) and *Xenopus laevis* (x) Atg4 orthologs. The alignment is based on the ClustalW algorithm. Note that isoforms A to D can be clearly separated in both organisms. **B**, Sequence alignment of human and *Xenopus laevis* Atg4B orthologs. Exchanges with regard to hsAtg4B are highlighted in yellow. Dark pink areas correspond to N- and C-terminal extensions based on the solved structures of human Atg4B. **C**, Phylogenetic tree of human and *Xenopus laevis* LC3 and GATE16 orthologs. Note that GATE16 forms a separate branch and can be clearly separated from the LC3 isoforms. **D and E**, Sequence alignment of human and *Xenopus laevis* LC3B and GATE16 orthologs, respectively. Exchanges with regard to the human proteins are highlighted in yellow. Mature human and *Xenopus laevis* GATE16 proteins share identical primary sequences.
(PDF)

S2 Fig Expression level and solubility of His_14_-UBL-tagged GFP. Proteins sketched in (**A**) were over-expressed in *E*. *coli* for 16 h at 18°C. Equal amounts of resuspended cells, total lysate and soluble material were analyzed by SDS-PAGE (**B**). GFP present in the soluble fraction was quantified via its absorbance at 488 nm. Note that scAtg8 promotes significantly lower expression levels than the other UBLs.
(PDF)

S3 Fig Cleavage standard. **A**, Schematic illustration of substrates xLC3B-MBP and xGATE16-MBP. **B**, Substrates xLC3B-MBP and xGATE16-MBP (200 μM) were individually incubated with 4 μM xAtg4B^14-384^ for 90 min at 30°C to achieve complete cleavage. Identical incubations were performed in the absence of the protease. Reactions were stopped by 20-fold dilution in SDS sample buffer followed by 5 min incubation at 95°C. Defined volumes of cleaved and uncleaved samples were mixed and resolved by SDS-PAGE and Coomassie staining. Small sketches on the right side indicate positions of the full-length substrates (fl) as well as the C- and N-terminal cleavage products (ccp and ncp, respectively). A faint band corresponding to the protease is indicated by an asterisk (*).
(PDF)

S4 Fig *In-vitro* assay for xAtg4B activity. Related to Figs 2C and 6A; side-by-side comparison of selected protease fragments. **A and B**, Protease titration at 0°C and 25°C, respectively. The substrates xLC3B-MBP or xGATE16-MBP (100 μM) were incubated for 1 h at 0°C (**A**) or 25°C (**B**) in the presence of defined concentrations of indicated proteases. Cleavage products were separated by SDS-PAGE and stained with Coomassie G250. Shown are full-length substrate proteins (fl) and the C-terminal cleavage products (ccp). For examples of complete gels see S5 Fig. **C**, Time course. 100 μM of xLC3B-MBP was incubated at 0°C with 500 nM of indicated protease fragments. At indicated time points, aliquots were withdrawn and analyzed as described before.
(PDF)

S5 Fig *In-vitro* assay for xAtg4B activity. Related to Fig 2C and S4 Fig; complete SDS-PAGE gels. **A and B**, Protease titration. The substrate xLC3B-MBP (100 μM) was incubated for 1 h at 0°C (**A**) or 25°C (**B**) in the presence of a defined concentrations of indicated proteases. Cleavage products were separated by SDS-PAGE and stained with Coomassie G250. Shown are full-length substrate proteins (fl) as well as C-terminal and N-terminal cleavage products (ccp and ncp, respectively). Bands marked with asterisk (*) correspond to the protease fragment. xAtg4B^25-384^ co-migrates with the C-terminal substrate cleavage product.
(PDF)

S6 Fig Binding of xAtg4B fragments to immobilized xLC3B and xGATE16. Related to Fig 3; complete SDS-PAGE gels. An equimolar mixture of full-length xAtg4B and indicated fragments (10 μM each) was incubated with immobilized xLC3B or xGATE16. A resin without bait protein (right panel) served as a specificity control. Bound proteins were analyzed by SDS-PAGE. xAtg4B degradation products lacking parts of the C-terminal extension are marked with an asterisk (*) in the input fractions. Note that binding is markedly reduced for protease fragments harboring C-terminal deletions. The pull-down efficiency is generally higher when using xLC3B instead of xGATE16 as a bait.
(PDF)

S7 Fig Long-term DLS measurement of xAtg4B^25-384^. DLS signals were acquired for ≈20 h while incubating xAtg4B^25-384^ at 37°C with protection from oxidation. Note that at this temperature the protease appears rather stable for ≈2 h. At longer incubation, a gradual increase in average particle size is observed, indicating slow denaturation and aggregate formation.
(PDF)

S8 Fig Temperature dependence and salt sensitivity of xAtg4B fragments. Related to Figs 6B and 6C; side-by-side comparison of selected protease fragments. **A**, Temperature dependence. Indicated xAtg4B fragments were incubated with 100 μM of xLC3B-MBP (left) or xGATE16-MBP (right) for 1 h at defined temperatures. Note that in comparison to the xGATE16-MBP substrate, twice as much protease was used for cleavage of the xLC3B-MBP substrate. **B**, Salt sensitivity. 100 μM of xLC3B-MBP (left) or xGATE16-MBP (right) were incubated for one hour at 0°C with 500 nM protease fragments at NaCl concentrations ranging from 0.2 to 1.5 M.
(PDF)

S9 Fig Cross-reactivity between recombinant tag-cleaving proteases. Related to Fig 8B; complete SDS-PAGE gels. 100 μM of substrate proteins were incubated with 20 μM of each protease for 3 h at 25°C. Cleavage products were separated by SDS-PAGE. Bands marked with an asterisk (*) originate from the added protease. For schematic representations of substrates see Fig 8A. Abbreviations: bd, *Brachypodium distachyon*; tr, *Triticum aestivum* (summer wheat).
(PDF)

S10 Fig Stability of UBL fusions in eukaryotic lysates. Related to Fig 9B; complete membrane stains and Western blots. **A**, Schematic representation of substrates used for (B). **B and C**, Stability of protease substrates in cell extracts. For experimental details see main text and Methods.
(PDF)

S11 Fig Stability of UBL fusions in *S*. *cerevisiae*. Related to Fig 9D; complete membrane stains and Western blots. **A**, Schematic representation of substrates used for expression in *S*. *cerevisiae* (B) harboring an N-terminal ZZ-tag, an ubiquitin-like protein (UBL) and a C-terminal Citrine. **B**, *In-vivo* stability of protease substrates in *S*. *cerevisiae*. Indicated protease substrates were over-expressed in a *S*. *cerevisiae* strain constitutively expressing H2B-CFP (see Methods). Total cell lysates were analyzed by Western blot with antibodies recognizing the ZZ-tag (middle panel) or Citrine and CFP (right panel). Equal loading was confirmed by staining the membrane after blotting (left panel).
(PDF)

S1 Table *E*. *coli* expression vectors. All *E*. *coli* expression vectors are low copy vectors (*ColE1* origin) conferring Kanamycin resistance.
(PDF)

S2 Table *S*. *cerevisiae* expression vectors. All yeast expression vectors (*2μ*, *URA3*) encode the respective protein under the control of the *GAL1* promoter.
(PDF)
