# Supplementary material for: The Xenopus laevis Atg4B Protease: Insights into Substrate Recognition and Application for Tag Removal from Proteins Expressed in Pro- and Eukaryotic Hosts
Source: PLoS One. 2015 Apr 29;10(4):e0125099. doi: 10.1371/journal.pone.0125099 (PMC4414272; doi:10.1371/journal.pone.0125099)
Supplement: S1 Fig — A, Phylogenetic tree of human (hs) and Xenopus laevis (x) Atg4 orthologs. The alignment is based on the ClustalW algorithm. Note that isoforms A to D can be clearly separated in both organisms. B, Sequence alignment of human and Xenopus laevis Atg4B orthologs. Exchanges with regard to hsAtg4B are highlighted in yellow. Dark pink areas correspond to N- and C-terminal extensions based on the solved structures of human Atg4B. C, Phylogenetic tree of human and Xenopus laevis LC3 and GATE16 orthologs. Note that GATE16 forms a separate branch and can be clearly separated from the LC3 isoforms. D and E, Sequence alignment of human and Xenopus laevis LC3B and GATE16 orthologs, respectively. Exchanges with regard to the human proteins are highlighted in yellow. Mature human and Xenopus laevis GATE16 proteins share identical primary sequences. (PDF) [file pone.0125099.s001.pdf]

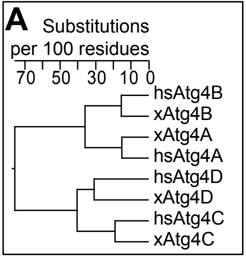

B Atg4B orthologs

```
hsAtg4B    1 MDAATLTYDTLRFAEFEDFPETSEPVWILGRKYSIFTEKDEILSDVASRLWFTYRKNFPAIGGTGPTSDTGWGCMLRCGQ 80
xAtg4B     1 MDAATLTYDTLRFADTPDFPETAEPVWVLGRKYSALTEKEQLLNDITSRLWFTYRRNFQAIGGTGPTSDTGWGCMLRCGQ 80

hsAtg4B   81 MIFAQALVCRHLGRDWRWTQRKRQPDSYFSVLNAFIDRKDSYYSIHQIAQMGVGEGKSIGQWYGPNTVAQVLKKLAVFDT 160
xAtg4B    81 MIFAQALICRHVGRDWRWDKQKPKGE-YLNILTAFLDKKDSYYSIHQIAQMGVGEGKYIGQWYGPNTVAQVLRKLAVFDQ 159

hsAtg4B  161 WSSLAVHIAMDNTVVMEEIRRLCRTSVPCAGATAFPADSDRHCNGFPAGAEVTNRPSPWRPLVLLIPLRLGLTDINEAYV 240
xAtg4B   160 WSSIAVHIAMDNTVVVDEIRRLCR------AGSGESSDAGALSNGYTGDSDPS--CAQWKPLVLLIPLRLGLSEINEAYI 231

hsAtg4B  241 ETLKHCFMMPQSLGVIGGKPNSAHYFIGYVGEELIYLDPHTTQPAVEPTDGCFIPDESFHCQHPPCRMSIAELDPSIAVG 320
xAtg4B   232 ETLKHCFMVPQSLGVIGGRPNSAHYFIGYVGDELIYLDPHTTQLSVEPSDCSFIEDESFHCQHPPCRMHVSEIDPSIAVG 311

hsAtg4B  321 FFCKTEDDFNDWCQQVKKLSLLGGALPMFELVELQPSHLACPDVLNLSLDSSDVERLERFFDSEDEDFEILSL 393
xAtg4B   312 FFCSSQEDFEDWCQHIKKLSLSGGALPMFEVVDQLPLHLSNPDVLNLTPDSSDADRLDRFFDSEDEEFEILSL 384
```

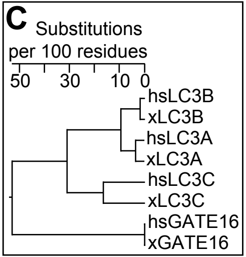

D LC3B orthologs

```
hsLC3B   1 MPSEKTFKQRRSFEQRVEDVRLIREQHPTKIPVIIERYKGEKQLPVLDKTKFLVPDHVNMSELIKIIRRRLQLNANQAFF 80
xLC3B    1 MPSEKTFKQRRSLEQRVEDVRLIREQHPTKIPVIIERYKGEKQLPVLDKTKFLVPDHVNMSELIKIIRRRLQLNSNQAFF 80

hsLC3B  81 LLVNGHSMVSVSTPISEVYESERDEDGFLYMVYASQETFG 120
xLC3B   81 LLVNGHSMVSVSTPISEVYEREKDEDGFLYMVYASQETFG 120
```

E GATE16 orthologs

```
hsGATE16  1 MKWMFKEDHSLEHRCVESAKIRAKYPDRVPVIVEKVSGSQIVDIDKRKYLVPSDITVAQFMWIIRKRIQLPSEKAIFLFV 80
xGATE16   1 MKWMFKEDHSLEHRCVESAKIRAKYPDRVPVIVEKVSGSQIVDIDKRKYLVPSDITVAQFMWIIRKRIQLPSEKAIFLFV 80

hsGATE16 81 DKTVPQSSLTMGQLYEKEKDEDGFLYVAYSGENTFG 116
xGATE16  81 DKTVPQSSLTMGQLYEKEKDEDGFLYVAYSGENTFG 116
```
