# Supplementary figures and images for: The Xenopus laevis Atg4B Protease: Insights into Substrate Recognition and Application for Tag Removal from Proteins Expressed in Pro- and Eukaryotic Hosts
Source: PLoS One. 2015 Apr 29;10(4):e0125099. doi: 10.1371/journal.pone.0125099 (PMC4414272; doi:10.1371/journal.pone.0125099)

**A**His<sub>14</sub>-UBL-GFP: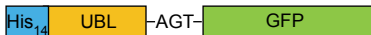**B**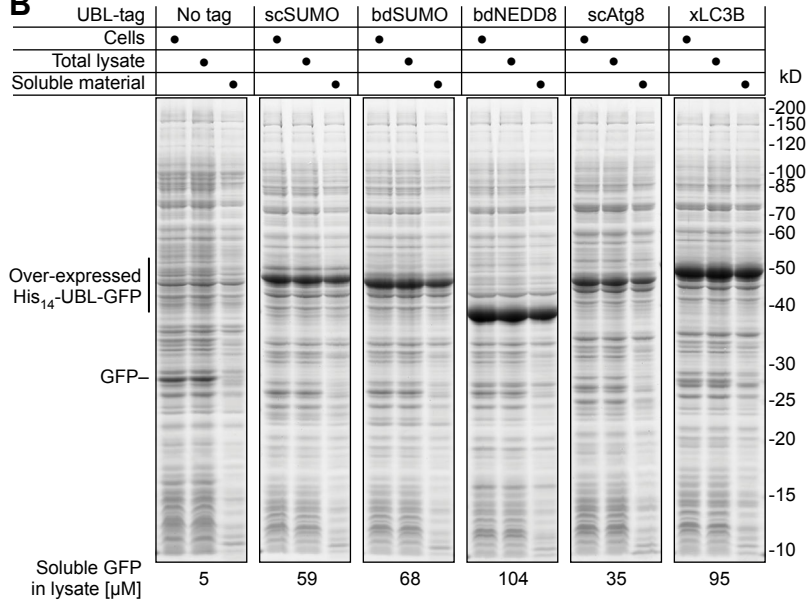

Supplement: S2 Fig — Proteins sketched in (A) were over-expressed in E. coli for 16 h at 18°C. Equal amounts of resuspended cells, total lysate and soluble material were analyzed by SDS-PAGE (B). GFP present in the soluble fraction was quantified via its absorbance at 488 nm. Note that scAtg8 promotes significantly lower expression levels than the other UBLs. (PDF) [file pone.0125099.s002.pdf]

**A**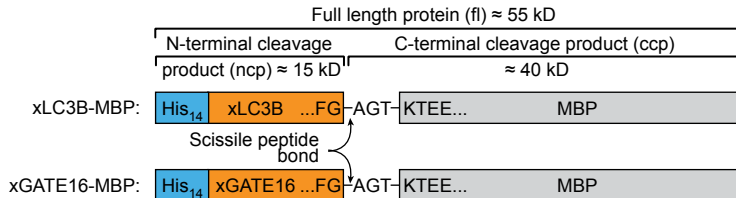**B**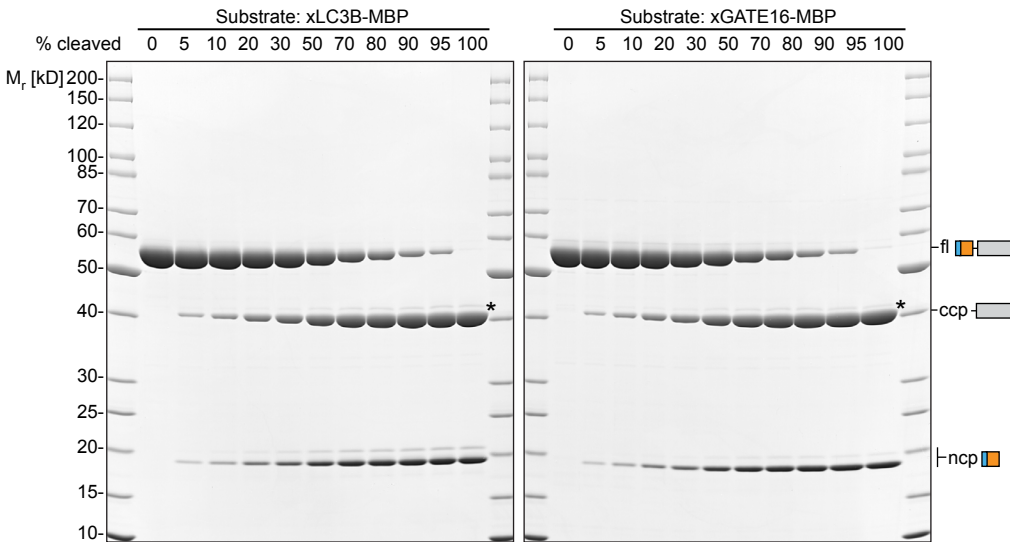

Supplement: S3 Fig — A, Schematic illustration of substrates xLC3B-MBP and xGATE16-MBP. B, Substrates xLC3B-MBP and xGATE16-MBP (200 μM) were individually incubated with 4 μM xAtg4B14-384 for 90 min at 30°C to achieve complete cleavage. Identical incubations were performed in the absence of the protease. Reactions were stopped by 20-fold dilution in SDS sample buffer followed by 5 min incubation at 95°C. Defined volumes of cleaved and uncleaved samples were mixed and resolved by SDS-PAGE and Coomassie staining. Small sketches on the right side indicate positions of the full-length substrates (fl) as well as the C- and N-terminal cleavage products (ccp and ncp, respectively). A faint band corresponding to the protease is indicated by an asterisk (*). (PDF) [file pone.0125099.s003.pdf]

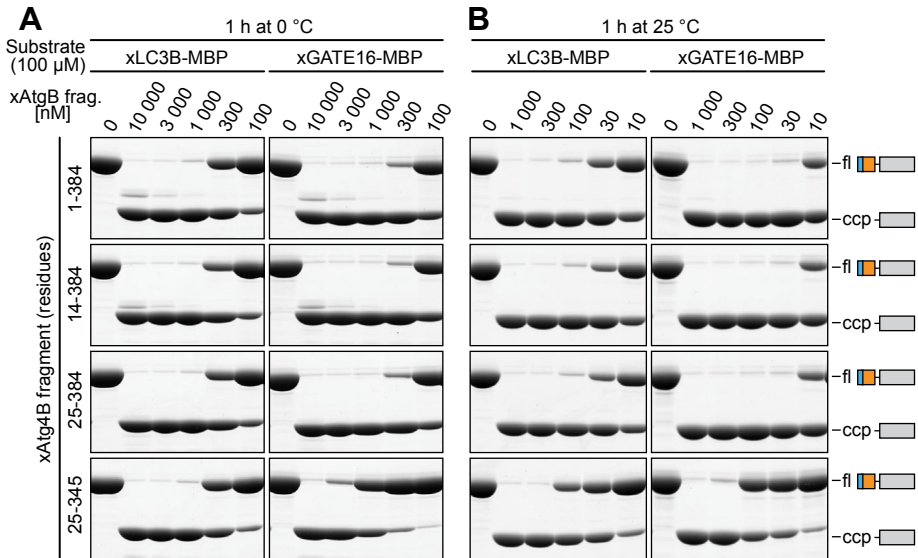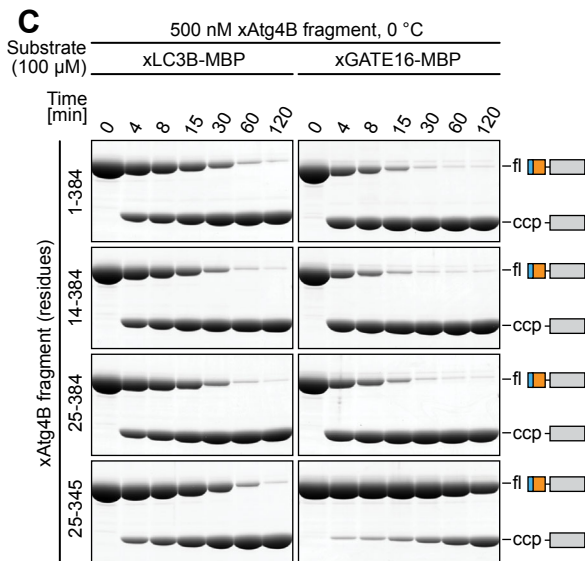

Supplement: S4 Fig — Related to Figs 2C and 6A; side-by-side comparison of selected protease fragments. A and B, Protease titration at 0°C and 25°C, respectively. The substrates xLC3B-MBP or xGATE16-MBP (100 μM) were incubated for 1 h at 0°C (A) or 25°C (B) in the presence of defined concentrations of indicated proteases. Cleavage products were separated by SDS-PAGE and stained with Coomassie G250. Shown are full-length substrate proteins (fl) and the C-terminal cleavage products (ccp). For examples of complete gels see S5 Fig. C, Time course. 100 μM of xLC3B-MBP was incubated at 0°C with 500 nM of indicated protease fragments. At indicated time points, aliquots were withdrawn and analyzed as described before. (PDF) [file pone.0125099.s004.pdf]

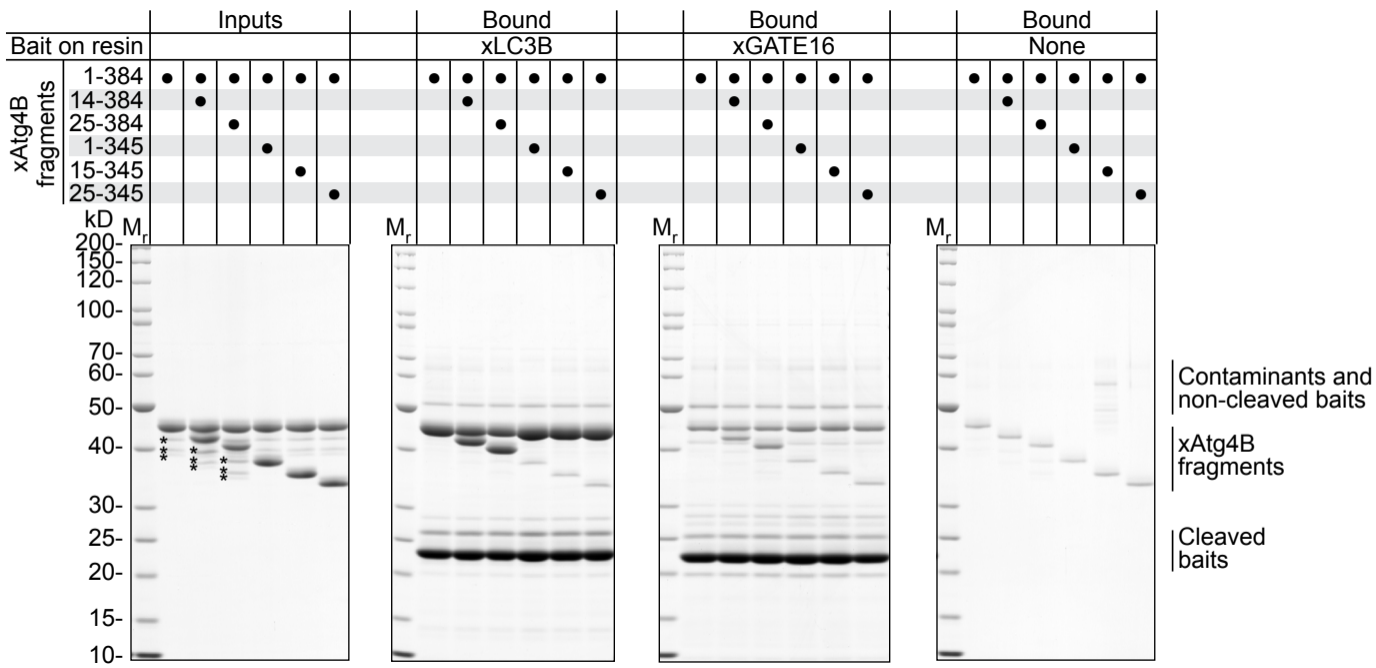

Supplement: S6 Fig — Related to Fig 3; complete SDS-PAGE gels. An equimolar mixture of full-length xAtg4B and indicated fragments (10 μM each) was incubated with immobilized xLC3B or xGATE16. A resin without bait protein (right panel) served as a specificity control. Bound proteins were analyzed by SDS-PAGE. xAtg4B degradation products lacking parts of the C-terminal extension are marked with an asterisk (*) in the input fractions. Note that binding is markedly reduced for protease fragments harboring C-terminal deletions. The pull-down efficiency is generally higher when using xLC3B instead of xGATE16 as a bait. (PDF) [file pone.0125099.s006.pdf]

—●— xAtg4B<sup>25-384</sup>

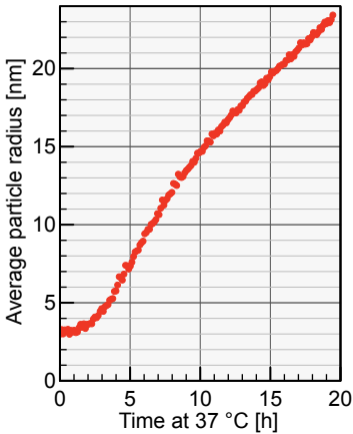

Supplement: S7 Fig — DLS signals were acquired for ≈20 h while incubating xAtg4B25-384 at 37°C with protection from oxidation. Note that at this temperature the protease appears rather stable for ≈2 h. At longer incubation, a gradual increase in average particle size is observed, indicating slow denaturation and aggregate formation. (PDF) [file pone.0125099.s007.pdf]

**A**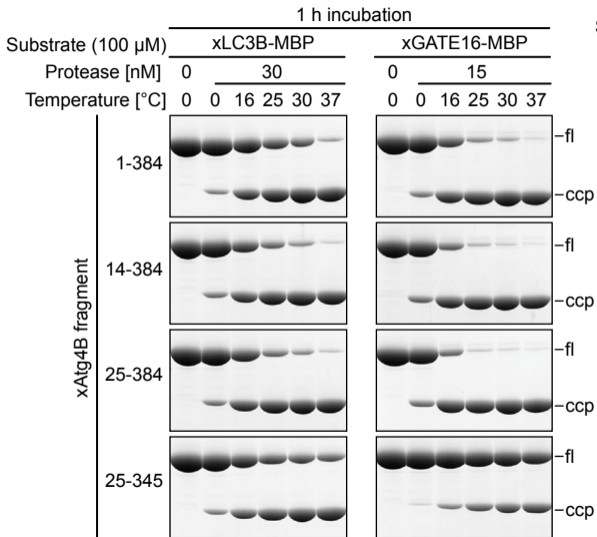**B**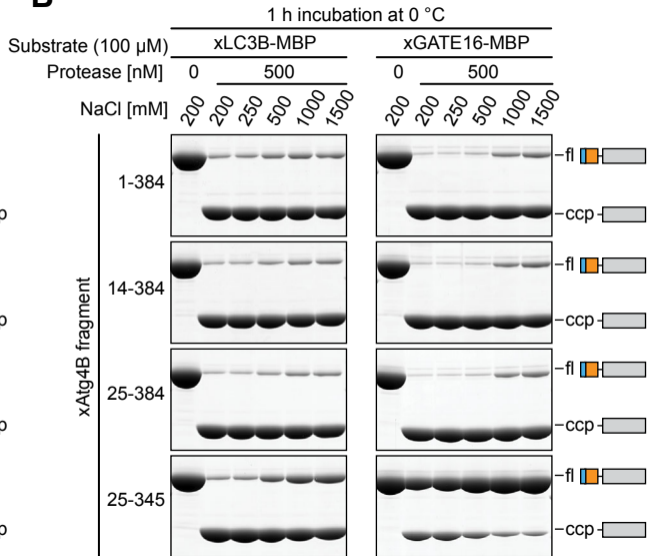

Supplement: S8 Fig — Related to Figs 6B and 6C; side-by-side comparison of selected protease fragments. A, Temperature dependence. Indicated xAtg4B fragments were incubated with 100 μM of xLC3B-MBP (left) or xGATE16-MBP (right) for 1 h at defined temperatures. Note that in comparison to the xGATE16-MBP substrate, twice as much protease was used for cleavage of the xLC3B-MBP substrate. B, Salt sensitivity. 100 μM of xLC3B-MBP (left) or xGATE16-MBP (right) were incubated for one hour at 0°C with 500 nM protease fragments at NaCl concentrations ranging from 0.2 to 1.5 M. (PDF) [file pone.0125099.s008.pdf]

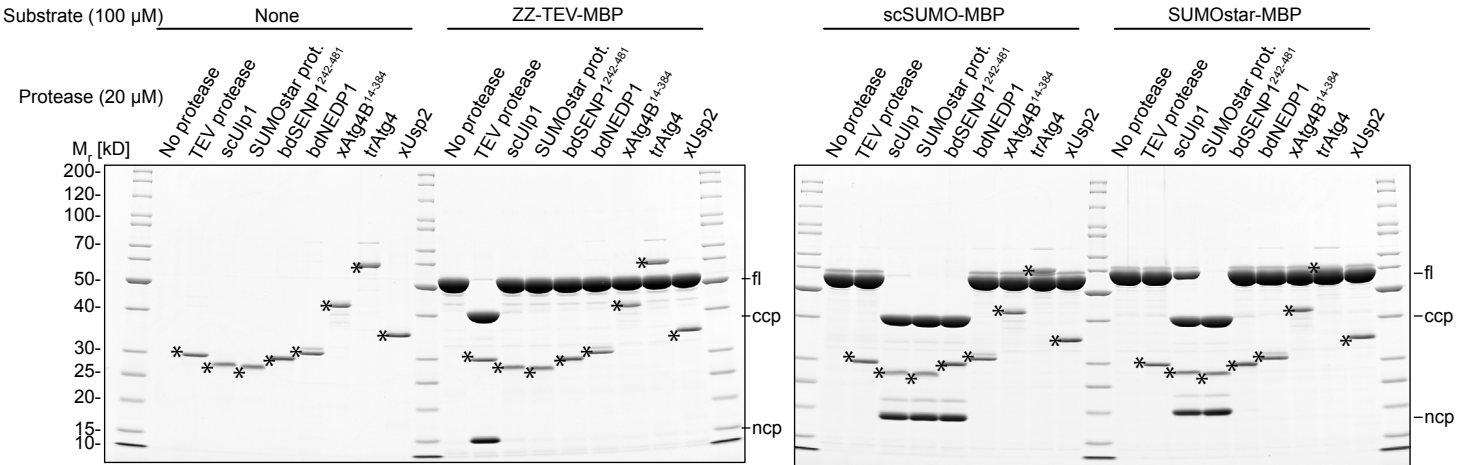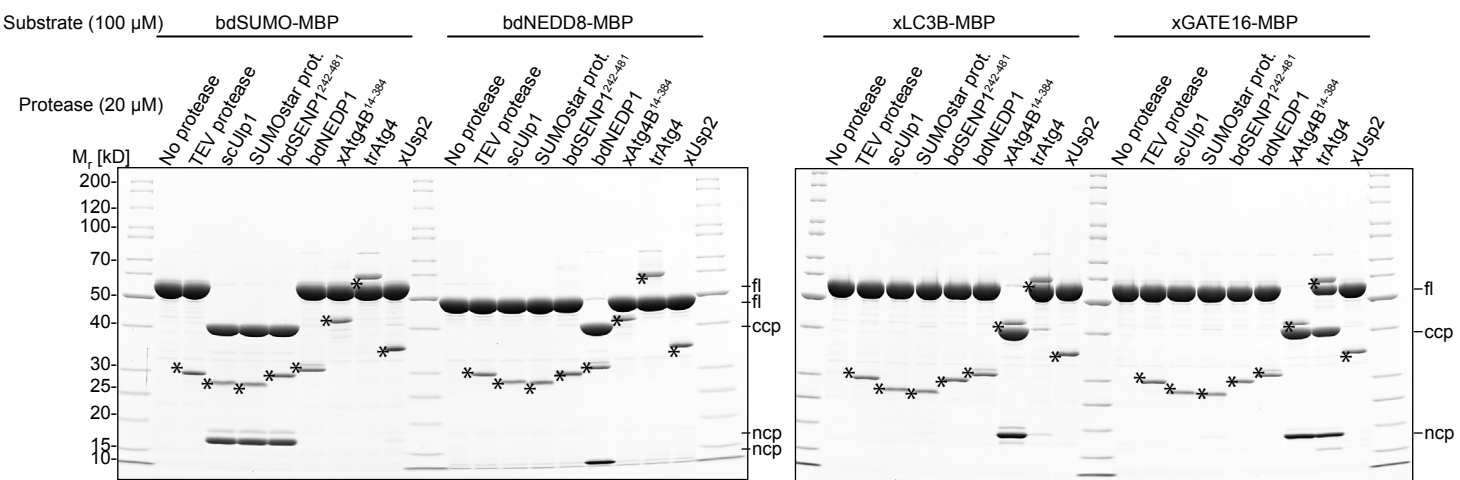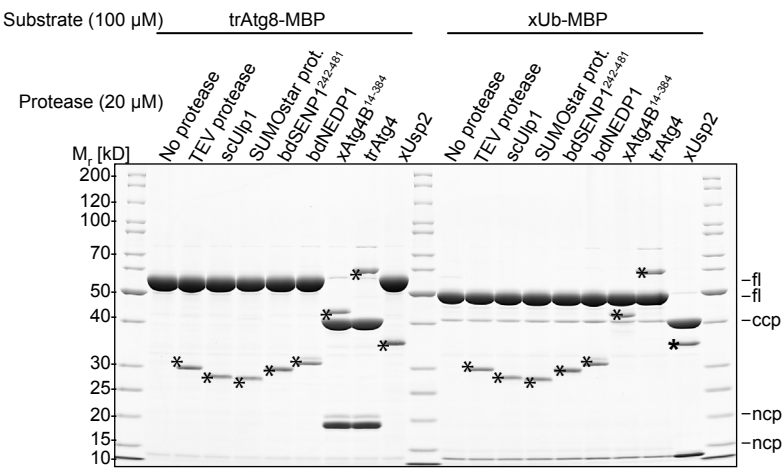

Supplement: S9 Fig — Related to Fig 8B; complete SDS-PAGE gels. 100 μM of substrate proteins were incubated with 20 μM of each protease for 3 h at 25°C. Cleavage products were separated by SDS-PAGE. Bands marked with an asterisk (*) originate from the added protease. For schematic representations of substrates see Fig 8A. Abbreviations: bd, Brachypodium distachyon; tr, Triticum aestivum (summer wheat). (PDF) [file pone.0125099.s009.pdf]

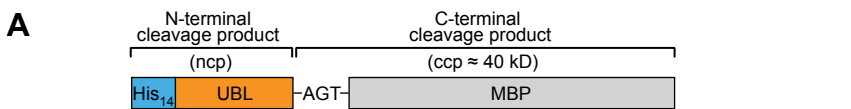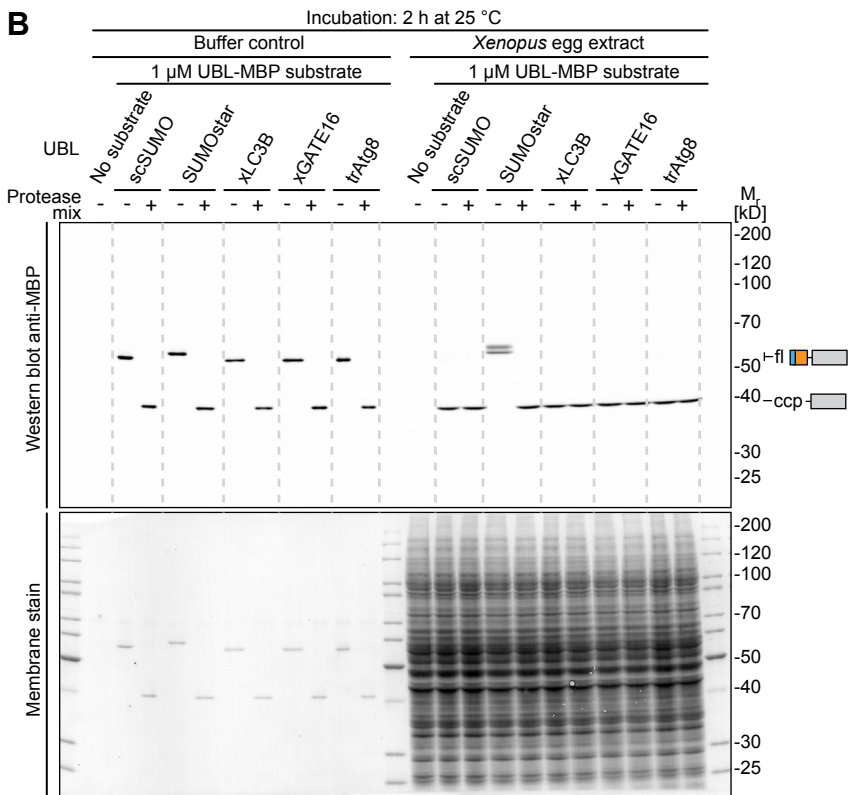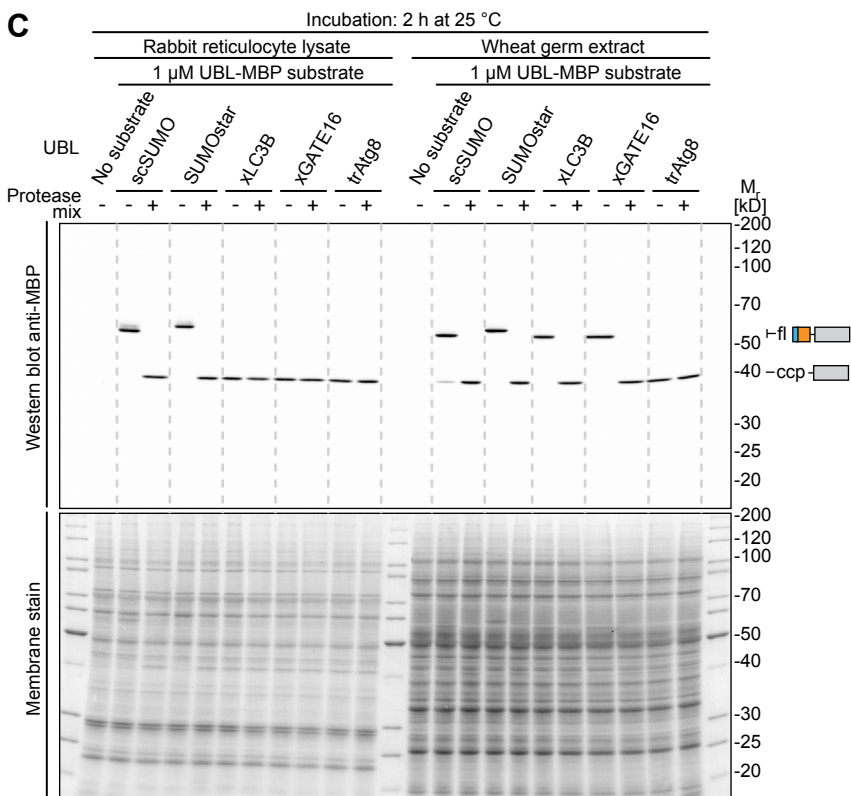

Supplement: S10 Fig — Related to Fig 9B; complete membrane stains and Western blots. A, Schematic representation of substrates used for (B). B and C, Stability of protease substrates in cell extracts. For experimental details see main text and Methods. (PDF) [file pone.0125099.s010.pdf]

**A**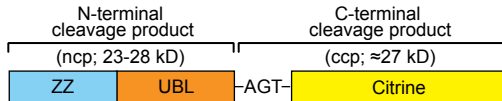**B**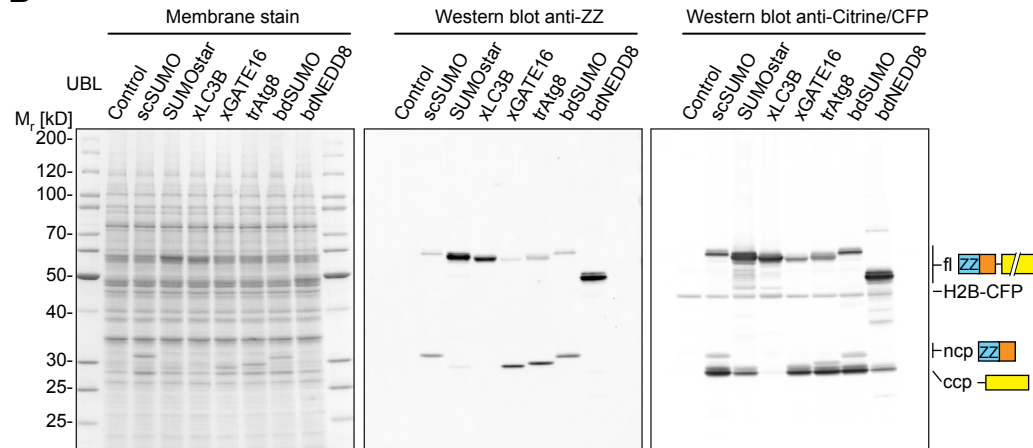

Supplement: S11 Fig — Related to Fig 9D; complete membrane stains and Western blots. A, Schematic representation of substrates used for expression in S. cerevisiae (B) harboring an N-terminal ZZ-tag, an ubiquitin-like protein (UBL) and a C-terminal Citrine. B, In-vivo stability of protease substrates in S. cerevisiae. Indicated protease substrates were over-expressed in a S. cerevisiae strain constitutively expressing H2B-CFP (see Methods). Total cell lysates were analyzed by Western blot with antibodies recognizing the ZZ-tag (middle panel) or Citrine and CFP (right panel). Equal loading was confirmed by staining the membrane after blotting (left panel). (PDF) [file pone.0125099.s011.pdf]
