# Supplementary material for: The Xenopus laevis Atg4B Protease: Insights into Substrate Recognition and Application for Tag Removal from Proteins Expressed in Pro- and Eukaryotic Hosts
Source: PLoS One. 2015 Apr 29;10(4):e0125099. doi: 10.1371/journal.pone.0125099 (PMC4414272; doi:10.1371/journal.pone.0125099)
Supplement: S5 Fig — Related to Fig 2C and S4 Fig; complete SDS-PAGE gels. A and B, Protease titration. The substrate xLC3B-MBP (100 μM) was incubated for 1 h at 0°C (A) or 25°C (B) in the presence of a defined concentrations of indicated proteases. Cleavage products were separated by SDS-PAGE and stained with Coomassie G250. Shown are full-length substrate proteins (fl) as well as C-terminal and N-terminal cleavage products (ccp and ncp, respectively). Bands marked with asterisk (*) correspond to the protease fragment. xAtg4B25-384 co-migrates with the C-terminal substrate cleavage product. (PDF) [file pone.0125099.s005.pdf]

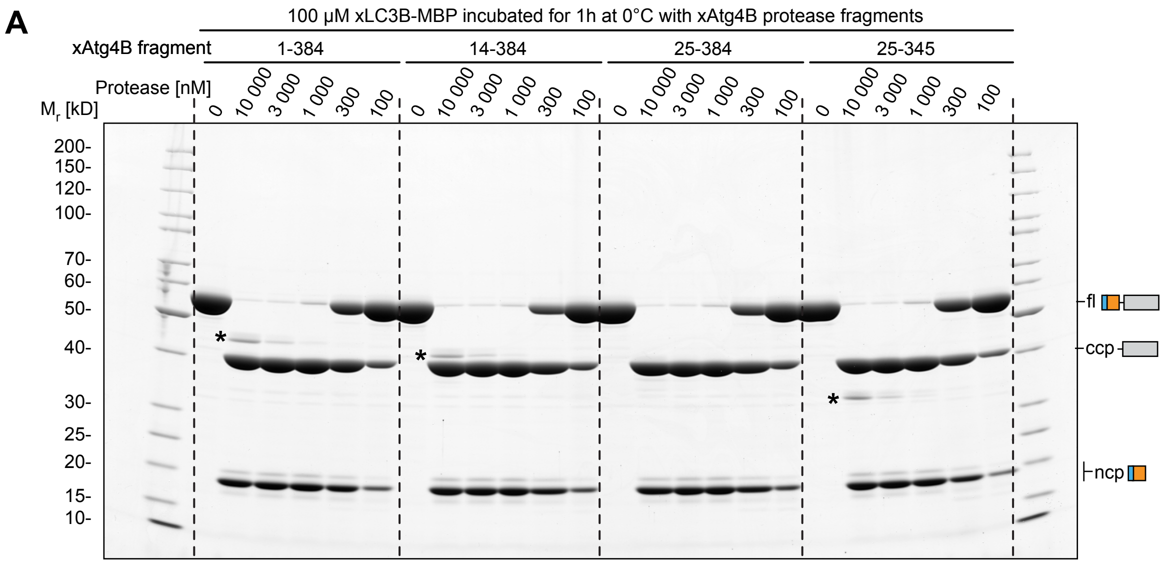
A
100 µM xLC3B-MBP incubated for 1h at 0°C with xAtg4B protease fragments
xAtg4B fragment
1-384
14-384
25-384
25-345
Protease [nM]
0 10 000 3 000 1 000 300 100
Mr [kD]
200-
150-
120-
100-
70-
60-
50-
40-
30-
25-
20-
15-
10-
fl
ccp
ncp

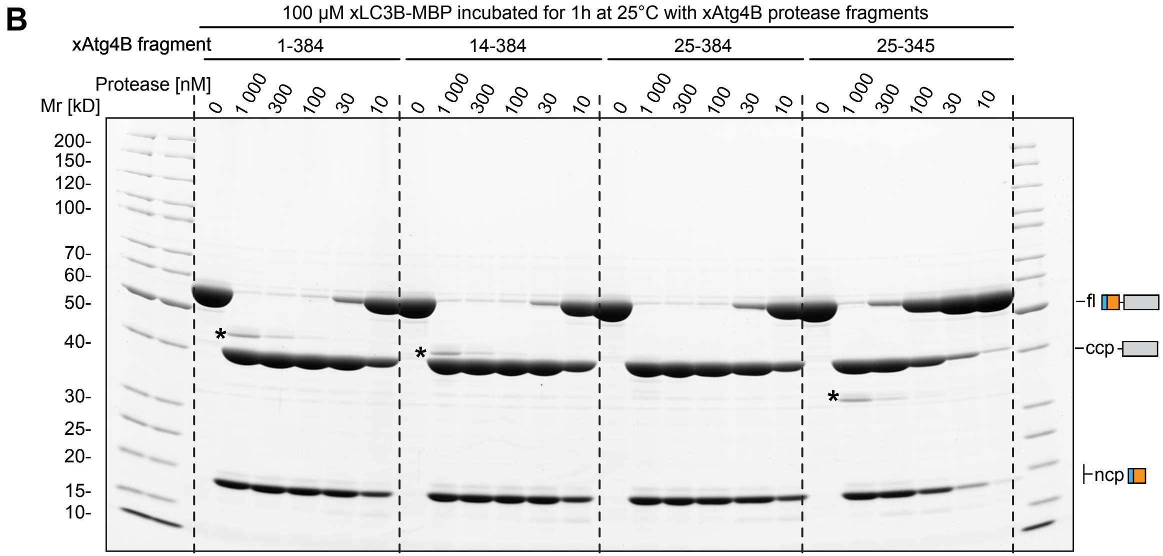
B
100 µM xLC3B-MBP incubated for 1h at 25°C with xAtg4B protease fragments
xAtg4B fragment
1-384
14-384
25-384
25-345
Protease [nM]
0 1 000 300 100 30 10
Mr [kD]
200-
150-
120-
100-
70-
60-
50-
40-
30-
25-
20-
15-
10-
fl
ccp
ncp
