# Supplementary material for: The Xenopus laevis Atg4B Protease: Insights into Substrate Recognition and Application for Tag Removal from Proteins Expressed in Pro- and Eukaryotic Hosts
Source: PLoS One. 2015 Apr 29;10(4):e0125099. doi: 10.1371/journal.pone.0125099 (PMC4414272; doi:10.1371/journal.pone.0125099)
Supplement: S1 Table — All E. coli expression vectors are low copy vectors (ColE1 origin) conferring Kanamycin resistance. (PDF) [file pone.0125099.s012.pdf]

S1 Table : *E. coli* expression vectors

Plasmid name	Expressed protein	Reference
pSF2542	His_{14}-TEV-xAtg4B	this study
pSF2319	His_{14}-TEV-xAtg4B$^{14\text{-}384}$	this study
pSF2401	His_{14}-TEV-xAtg4B$^{25\text{-}384}$	this study
pSF2566	His_{14}-TEV-xAtg4B$^{1\text{-}345}$	this study
pSF2402	His_{14}-TEV-xAtg4B$^{14\text{-}345}$	this study
pSF2403	His_{14}-TEV-xAtg4B$^{25\text{-}345}$	this study
pSF2317	His_{14}-xLC3B-MBP	this study
pSF2530	His_{14}-xGATE16-MBP	this study
pSF2376	His_{14}-xLC3B-MGT-MBP	this study
pSF2377	His_{14}-xLC3B-YGT-MBP	this study
pSF2378	His_{14}-xLC3B-EGT-MBP	this study
pSF2379	His_{14}-xLC3B-RGT-MBP	this study
pSF2380	His_{14}-xLC3B-PGT-MBP	this study
pSF2089	His_{14}-brNEDD8-mCherry	this study
pSF2555	His_{14}-IF2d1-xLC3B-mEGFP	this study
pSF2559	His_{14}-IF2d1-xGATE16-mEGFP	this study
pSF2563	His_{14}-IF2d1-xLC3B-MBP	this study
pSF2562	His_{14}-IF2d1-xGATE16-MBP	this study
pSF2528	His_{14}-Spacer-xLC3B-mEGFP	this study
pSF2558	His_{14}-Spacer-xGATE16-mEGFP	this study
pSF2075	His_{10}-ZZ-TEV-GT-MBP	[33]
pSF1476	His_{14}-scSUMO-MBP	[33]
pSF1477	His_{14}-bdSUMO-MBP	[33]
pSF1478	His_{14}-bdNEDD8-MBP	[33]
pSF2293	His_{14}-trAtg8-MBP	this study
pSF2240	His_{14}-xUb-MBP	[33]
pSF2176	His_{14}-scAtg8-MBP	[33]
pSF2541	His_{14}-SUMOstar-MBP	this study
pSF590	MBP-TEV-His_{14}-TEV(SH)ΔC6	[33]
pSF1389	His_{14}-TEV-bdSENP1$^{242\text{-}481}$	[33]
pSF1877	His_{14}-TEV-scUlp1	[33]
pSF1878	His_{14}-TEV-SUMOstar protease	this study
pSF1443	His_{14}-TEV-bdNEDP1	[33]
pSF2179	His_{14}-bdSUMO-scAtg4	[33]
pSF2295	His_{14}-TEV-trAtg4	this study
pSF2241	His_{14}-bdSUMO-xUsp2$^{43\text{-}463}$	[33]
pSF2367	mEGFP	this study
pSF2343	His_{14}-scSUMO-mEGFP	this study
pSF2344	His_{14}-bdSUMO-mEGFP	this study
pSF2345	His_{14}-bdNEDD8-mEGFP	this study
pSF2347	His_{14}-scAtg8-mEGFP	this study
pSF2350	His_{14}-xLC3B-mEGFP	this study

All *E. coli* expression vectors are low copy vectors (*ColE1* origin) conferring Kanamycin resistance.
