# Supplementary material for: The Xenopus laevis Atg4B Protease: Insights into Substrate Recognition and Application for Tag Removal from Proteins Expressed in Pro- and Eukaryotic Hosts
Source: PLoS One. 2015 Apr 29;10(4):e0125099. doi: 10.1371/journal.pone.0125099 (PMC4414272; doi:10.1371/journal.pone.0125099)
Supplement: S2 Table — All yeast expression vectors (2μ, URA3) encode the respective protein under the control of the GAL1 promoter. (PDF) [file pone.0125099.s013.pdf]

S2 Table: *S. cerevisiae* expression vectors

Plasmid name	Expressed protein	Reference
pSF2305	ZZ-scSUMOstar-Citrine	this study
pSF2551	ZZ-xLC3B-Citrine	this study
pSF2552	ZZ-xGATE16-Citrine	this study
pSF2553	ZZ-trAtg8-Citrine	this study
pSF2554	ZZ-scSUMO-Citrine	this study
pSF2565	ZZ-bdSUMO-Citrine	this study
pSF2564	ZZ-bdNEDD8-Citrine	this study

All yeast expression vectors (*2µ, URA3*) encode the respective protein under the control of the *GAL1* promoter.
